# Rigid reduced successor representation as a potential mechanism for addiction

**DOI:** 10.1111/ejn.15227

**Published:** 2021-05-10

**Authors:** Kanji Shimomura, Ayaka Kato, Kenji Morita

**Affiliations:** ^1^ Physical and Health Education Graduate School of Education The University of Tokyo Tokyo Japan; ^2^ Department of Behavioral Medicine National Institute of Mental Health National Center of Neurology and Psychiatry Kodaira Japan; ^3^ Department of Life Sciences Graduate School of Arts and Sciences The University of Tokyo Tokyo Japan; ^4^ Laboratory for Circuit Mechanisms of Sensory Perception RIKEN Center for Brain Science Wako Japan; ^5^ Research Fellowship for Young Scientists Japan Society for the Promotion of Science Tokyo Japan; ^6^ International Research Center for Neurointelligence (WPI‐IRCN) The University of Tokyo Tokyo Japan

**Keywords:** addiction, dopamine, habit, reward prediction error, spiral striatum‐midbrain circuit

## Abstract

Difficulty in cessation of drinking, smoking, or gambling has been widely recognized. Conventional theories proposed relative dominance of habitual over goal‐directed control, but human studies have not convincingly supported them. Referring to the recently suggested “successor representation (SR)” of states that enables partially goal‐directed control, we propose a dopamine‐related mechanism that makes resistance to habitual reward‐obtaining particularly difficult. We considered that long‐standing behavior towards a certain reward without resisting temptation can (but not always) lead to a formation of rigid dimension‐reduced SR based on the goal state, which cannot be updated. Then, in our model assuming such rigid reduced SR, whereas no reward prediction error (RPE) is generated at the goal while no resistance is made, a sustained large positive RPE is generated upon goal reaching once the person starts resisting temptation. Such sustained RPE is somewhat similar to the hypothesized sustained fictitious RPE caused by drug‐induced dopamine. In contrast, if rigid reduced SR is not formed and states are represented individually as in simple reinforcement learning models, no sustained RPE is generated at the goal. Formation of rigid reduced SR also attenuates the resistance‐dependent decrease in the value of the cue for behavior, makes subsequent introduction of punishment after the goal ineffective, and potentially enhances the propensity of nonresistance through the influence of RPEs via the spiral striatum‐midbrain circuit. These results suggest that formation of rigid reduced SR makes cessation of habitual reward‐obtaining particularly difficult and can thus be a mechanism for addiction, common to substance and nonsubstance reward.

## Graphical abstract

Long‐standing behavior towards a certain reward can lead to a formation of rigid reduced successor representation (SR) of states based on the future occupancy of the rewarded goal state. If it is formed, whereas no reward prediction error (RPE) is generated at the goal while the person does not resist temptation, once the person starts resistance, a sustained large RPE is generated at the goal, somewhat similarly to the hypothesized sustained fictitious RPE caused by drug‐induced dopamine.

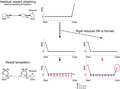

**Abbreviations**
DA: dopamine
RL: reinforcement learning
RPE: reward prediction error
SR: successor representation
TD: temporal‐difference

## 1 INTRODUCTION

Cessation of habitual drinking, smoking, gambling, or gaming can be quite difficult even with strong intention. Reasons for this, and whether there are reasons common to substance and nonsubstance reward, remain elusive. Although much effort has been devoted to developing clinical programs including technology‐based therapies (e.g., Gustafson et al., 2014; Kato et al., 2020; reviewed in Newman et al., 2011; Haskins et al., 2017), the lack of mechanistic understanding of the undesired addictive habit is an obstacle for further improvement. Computational modeling has become a powerful approach to elucidating the mechanisms of psychiatric disorders including addiction (Huys et al., 2016; Kato et al., 2020; Montague et al., 2012; Wang & Krystal, 2014). However, it appears that relatively less focus has been given to nonsubstance, compared to substance, addiction, although there have been proposals (e.g., Ognibene et al., 2019; Piray et al., 2010; Redish et al., 2007). In the present study, we explored possible computational and neural circuit mechanisms for why resisting habitual reward‐obtaining behavior can be quite difficult, with the following four streams of findings and suggestions in mind:

### 1.1 Involvement of the dopamine system in both substance and nonsubstance addiction

The dopamine (DA) system has been suggested to be crucially involved in substance addiction (Berke & Hyman, 2000), possibly through drug‐induced DA acting as a fictitious RPE that cannot be canceled out by predictions (Keiflin & Janak, 2015; Redish, 2004). However, there have also been implications of possible involvements of the DA system in nonsubstance addiction (Grant et al., 2010). Specifically, possible relations of medicines of Parkinson disease to pathological gambling (Dodd et al., 2005; Voon et al., 2006), as well as similar changes in the DA system in addiction to substance and nonsubstance such as game (Thalemann et al., 2007) or internet (Hou et al., 2012), have been suggested.

### 1.2 Goal‐directed and habitual behavior and their neural substrates, and their relations to addiction

It has been suggested that there are two behavioral processes, namely, goal‐directed and habitual behavior, which are sensitive or insensitive to changes in outcome values and/or action‐outcome contingencies, respectively (Balleine & Dickinson, 1998; Balleine & O'Doherty, 2010; Dolan & Dayan, 2013). They are suggested to be hosted by distinct corticostriatal circuits, specifically, those including ventral/dorsomedial striatum (or caudate) and those including dorsolateral striatum (or putamen), respectively (Corbit et al., 2001; Yin et al., 2004, 2005), where ventral‐to‐dorsal spiral influences have been anatomically suggested (Haber et al., 2000; Joel & Weiner, 2000). Computationally, goal‐directed and habitual behavior have been suggested to correspond to model‐based reinforcement learning (RL) and model‐free RL, respectively (Daw et al., 2005; but see Dezfouli & Balleine, 2012, for a critique of model‐free RL as a model of habitual behavior). It has been suggested that addiction can be caused by impaired goal‐directed and/or excessive habitual control (Everitt & Robbins, 2005, 2016). This is supported by multitudes of animal experiments, and there also exist findings in humans in line with this (Gillan et al., 2016). However, it has also been shown that human addicts often show goal‐directed behavior, such as those sensitive to outcome devaluation (Hogarth et al., 2019), although there are mixed results (as reviewed in Hogarth et al., 2019) and sensitivity can also differ between appetitive and aversive outcomes as shown for cocaine addiction (Ersche et al., 2016). Also, there have been proposals of many different possible causes for addiction (Redish et al., 2007), including those related to the way of state representation (Redish et al., 2008), hierarchical organization of learning systems (Keramati & Gutkin, 2013), homeostatic RL (Keramati et al., 2017), or limitations of cognitive resources and costs of exploration for both model‐based and model‐free systems (Ognibene et al., 2019).

### 1.3 Intermediate of goal‐directed and habitual behavior through successor representation of states

A great mystery had been that how model‐based and model‐free RLs, whose typical algorithms are so different in formulae, can be both hosted by corticostriatal‐DA circuits, different parts of which should still share basic architectures. Recent work (Gershman, 2018; Russek et al., 2017) has provided a brilliant potential solution to this by proposing that certain types of goal‐directed (model‐based) behavior, having sensitivity to changes in outcome values, can be achieved through a particular type of state representation called the successor representation (SR) (Dayan, 1993), combined with the ever‐suggested representation of RPE by DA (Montague et al., 1996; Schultz et al., 1997). In the SR, individual states are represented by a sort of closeness to their successor states, or more accurately, by time‐discounted cumulative future occupancies of these states. Behavior based on this representation is not fully goal‐directed, having difficulty in revaluation of state transition or policy, which has been demonstrated in actual human behavior (Momennejad et al., 2017) referred to as “subtler, more cognitive notion of habit” by the authors (Momennejad et al., 2017). SR and value update based on it have been suggested to be implemented in the prefrontal/hippocampus‐dorsomedial/ventral striatum circuits (Garvert et al., 2017; Russek et al., 2017; Stachenfeld et al., 2017), while circuits including dorsolateral striatum might implement habitual or model‐free behavior through “punctate” (i.e., individual) representation of states or actions.

### 1.4 Sustained DA response to predictable reward, possibly related to state representation

The original experiments that led to the proposal of representation of RPE by DA (Montague et al., 1996; Schultz et al., 1997) have shown that DA response to reward disappears after monkeys repeatedly experienced the stimulus(‐action)‐reward association and the reward presumably became predictable for them. However, sustained, and often ramping, DA signals to/towards (apparently) predictable reward has been widely observed in recent years (Collins et al., 2016; Guru et al., 2020; Hamid et al., 2019; Hamid et al., 2016; Howe et al., 2013; Kim et al., 2019; Mohebi et al., 2019; Sarno et al., 2020). There are a number of possible accounts for such sustained DA signals, positing that they represent RPE (Gershman, 2014; Kato & Morita, 2016; Kim et al., 2019; Mikhael et al., 2019; Morita & Kato, 2014; Song & Lee, 2020) or something different from RPE (Guru et al., 2020; Hamid et al., 2019; Hamid et al., 2016; Howe et al., 2013; Mohebi et al., 2019; Sarno et al., 2020) or both (Collins et al., 2016; Lloyd & Dayan, 2015). Of particular interest to our present work, one hypothesis (Gershman, 2014) suggests that sustained (ramping) DA signals might represent sustained RPE generated due to imperfect approximation of value function in the system using representation of states by low‐dimensional features.

Referring to these different streams of findings and suggestions, we propose a computational explanation on why resisting habitual reward‐obtaining can become particularly difficult.

## 2 MATERIALS AND METHODS

### 2.1 States, actions, policies, temporal discounting, and addicted/nonaddicted cases

**FIGURE 1:**
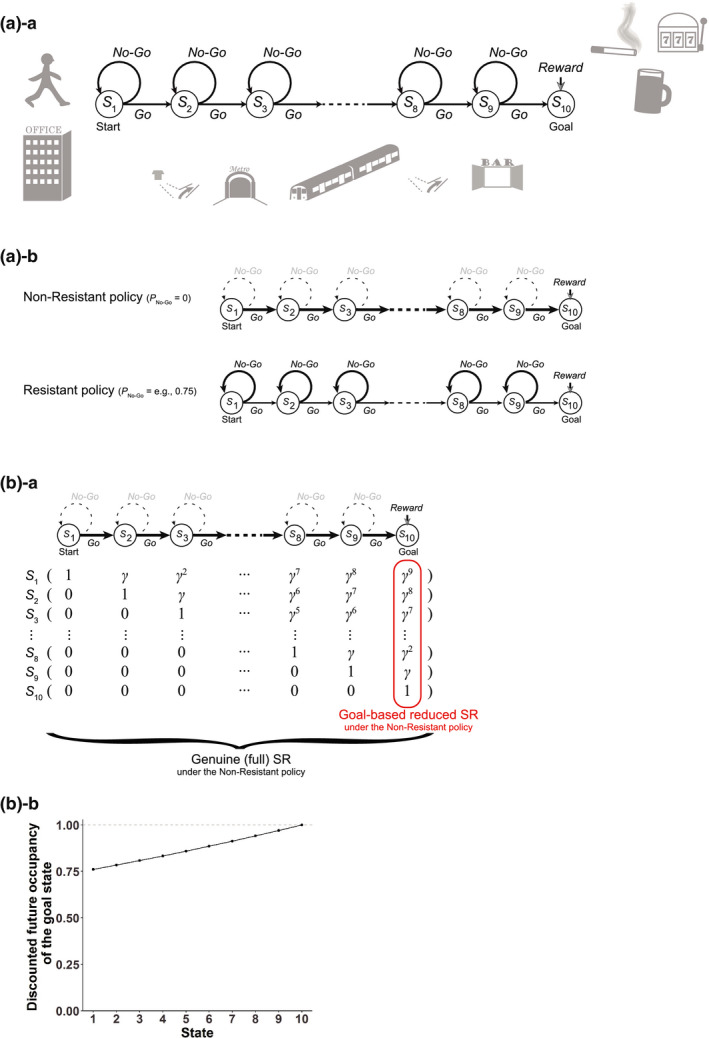
Schematic diagram of the model and the assumed goal‐based reduced successor representation (SR) of states under the Non‐Resistant policy. (a)‐a Schematic diagram of the model, adapted, with alterations, from figure 1 of Kato and Morita (2016). (a)‐b The Non‐Resistant policy, in which only “Go” action is chosen, and the Resistant policy, in which not only “Go” but also “No‐Go” action is chosen with a certain probability (*P*_No‐Go_). (b)‐a Genuine (full) SR, in which every state is represented by the discounted future occupancies of all the states, and the goal‐based reduced SR, in which every state is represented by the discounted future occupancy of only the final successor state, i.e., the goal state, both under the Non‐Resistant policy. *γ* indicates the time discount factor. (b)‐b The vertical axis indicates the discounted future occupancy of the goal state for each state (corresponding to the scalar feature of the state in the goal‐based reduced SR), given by *x*(*S_k_*) = *γ* ^10−^ *^k^* (Equation 6) for state *S_k_* (*k* = 1, …, 10; *S*_1_ is the start state and *S*_10_ is the goal state) with *γ* set to 0.97

We considered a series of states *S_k_* (*k* = 1, …, *n*; *S*_1_ is the start state and *S_n_* is the goal state) and actions “Go” and “No‐Go” as shown in  Figure 1(a)‐a. At the goal, reward *R*, whose size was set to 1, was assumed to be obtained. We considered two policies: the Non‐Resistant policy, in which the agent always takes “Go”, and the Resistant policy, in which the agent takes “No‐Go” with a certain probability (*P*_No‐Go_). *P*_No‐Go_ was mainly set to 0.75, with 0.5 and 0.9 also tested in simulations shown in Figure 4(a) and the Figures S1–S3. Under the Non‐Resistant policy, the state value of each state is calculated as follows:

(1) $$V_{\text{Non-Resistant}}\left(S_{k}\right)=R\gamma^{n‐k},$$

where *γ* is the time discount factor. The number of states from the start state to the goal state (*n*) was set to 10, and the time discount factor (*γ*) was mainly set to 0.97, with 0.95 and 0.99 also tested in simulations shown in the Figures S1–S3. This resulted in that the value at the start state was 0.97^9^ (≈0.76), or 0.95^9^ (≈0.63) or 0.99^9^ (≈0.91), times of the value at the goal. We assumed 10 states because it seems intuitively reasonable to assume that the long‐standing daily behavior to obtain a particular reward, such as going to a favorite pub for a beer after work, consists of around several to 10 distinct actions, for example, clean the desktop, wear the jacket, wait for and get on the elevator, walk to the subway station, wait for and get on a train, walk to the pub, call the waitstaff, and order the beer. These series of actions would typically take dozens to tens of minutes. Given this, we determined the abovementioned range of time discount factor in reference to a study (Buono et al., 2017), which examined temporal discounting for video gaming and found that the subjective value of video gaming 1 hr later was on average around 0.65–0.8 times of the value of immediate video gaming. Notably, however, the temporal discounting reported in that study appears to have near flat tails, indicating that it would not be well approximated by exponential functions, whereas we assumed exponential discounting.

We considered that if a person has long been taking a series of actions leading to a certain reward without resisting temptation (i.e., taking the Non‐Resistant policy), a reduced SR of states based on the goal state (explained below) have potentially been formed so rigidly that it cannot be updated after the person changes the policy. We tentatively refer to the case with formation of such rigid reduced SR as the addicted case, and other case as the nonaddicted case; at the beginning of Section 4, we will discuss the rationale for this naming.

### 2.2 Simple RL model with individual state representation, simulating the nonaddicted case

We considered a simple RL model with individual (or “punctate”) state representation to simulate the nonaddicted case. We assumed that each state has its own estimated state value, *V*_simple_(*S_k_*), and it is updated using (temporal‐difference(TD)‐type) RPE *δ*_simple_ at every time step:

(2) $$\delta_{\text{simple}}=R\left(S\left(t\right)\right)+\gamma V_{\text{simple}}\left(S\left(t+1\right)\right)‐V_{\text{simple}}\left(S\left(t\right)\right),$$

where *S*(*t*) and *S*(*t* + 1) are the states at time *t* and *t* + 1, respectively, and if *S*(*t*) is the goal state, the term *γV*_simple_(*S*(*t* + 1)) is dropped, except for in simulations where punishment was considered (described below). *R*(*S*(*t*)) is the reward value obtained at *S*(*t*), which was assumed to be 0 except for the goal state, except for in simulations where punishment was considered. RPE upon initiation of behavior was assumed to be:

(3) $$0+\gamma V_{\text{simple}}\left(S_{1}\right)‐0=\gamma V_{\text{simple}}\left(S_{1}\right).$$

*V*_simple_ was assumed to be updated as follows:

(4) $$V_{\text{simple}}\left(S\left(t\right)\right)\to V_{\text{simple}}\left(S\left(t\right)\right)+\alpha_{\text{simple}}\delta_{\text{simple}},$$

where *α*_simple_ is the learning rate, which was set to 0.5 unless otherwise mentioned. For simulations of behavior under the Non‐Resistant policy, initial values of each state value *V*_simple_(*S_k_*) were set to 0. For simulations of behavior under the Resistant policy, initial values of each state value were set to the values corresponding to the completion of learning under the Non‐Resistant policy, specifically,

(5) $$V_{\text{simple}}\left(S_{k}\right)=V_{\text{Non-Resistant}}\left(S_{k}\right)=R\gamma^{n‐k}.$$

We also simulated the cases where punishment (negative reward) is introduced in a state following the goal state. Specifically, for these simulations, we additionally assumed state *S*_11_ which is the next state of the goal state *S*_10_. At *S*_10_, *S*(*t* + 1) in Equation (2) was assumed to be *S*_11_, and at *S*_11_, *R*(*S*_11_) was set to −2 and the term *γV*_simple_(*S*(*t* + 1)) in Equation (2) was dropped. The initial condition corresponding to the completion of learning under the Non‐Resistant policy without punishment, that is, *V*_simple_(*S_k_*) = *γ* ^10−^ *^k^* for *k* = 1, …, 10 and *V*(*S*_11_) = 0, was assumed, and the agent's behavior under the Non‐Resistant policy with punishment was simulated.

### 2.3 Model with rigid goal‐based reduced SR of states, simulating the addicted case

We considered a model with rigid goal‐based reduced SR of states to simulate the addicted case. Specifically, we considered a single (i.e., scalar) feature *x* and assumed that the *k*‐th state, *S_k_* (*k* = 1, …, *n*; *S*_1_ is the start state and *S_n_* is the goal state), is represented by

(6) $$x\left(S_{k}\right)=\gamma^{n‐k}.$$

We assumed that the agent estimates the (true) state value of each state under a given policy by a linear function of these scalar features with a coefficient *w*:

(7) $$V_{\text{policy}}\left(S_{k}\right)\approx wx\left(S_{k}\right),$$

The (true) state value under the Non‐Resistant policy (Equation 1) is in fact exactly obtained as a linear function of these scalar features with *w* equal to the reward value obtained at the goal (*R*):

(8) $$V_{\text{Non-Resistant}}\left(S_{k}\right)=R\gamma^{n‐k}=Rx\left(S_{k}\right).$$

We assumed that starting from this condition (*w* = *R*), which corresponds to the completion of learning under the Non‐Resistant policy, the agent learns (estimates) the (true) state value under the Resistant policy by updating the coefficient *w* using (TD‐type) RPE *δ*_RSR_ at every time step:

(9) $$\delta_{\text{RSR}}=R\left(S\left(t\right)\right)+\gamma wx\left(S\left(t+1\right)\right)‐wx\left(S\left(t\right)\right),$$

where if *S*(*t*) is the goal state, the term *γwx*(*S*(*t* + 1)) is dropped. Specifically, *w* was assumed to be updated as follows:

(10) $$w\to w+\alpha_{\mathrm{RSR}}x\left(S\left(t\right)\right)\delta_{\mathrm{RSR}},$$

where *α*_RSR_ is the learning rate, which was set to 0.5 unless otherwise mentioned. This way of linear function approximation and RPE‐based update (Sutton, 1988; Sutton & Barto, 2018) has been typically assumed in neuro‐computational models and is considered to be implementable through synaptic plasticity depending on DA, which represents RPE, and presynaptic activity, which represents *x*(*S*(*t*)) (Montague et al., 1996; Russek et al., 2017). The initial value of *w* was set to *R* (=1), with which the approximate value function exactly matches the true value function under the Non‐Resistant policy (as mentioned above). RPE upon initiation of behavior was assumed to be:

(11) $$0+\gamma wx\left(S_{1}\right)‐0=w\gamma^{n}.$$

Notably, for the model with rigid reduced SR, we did not conduct simulation for the person's behavior under the Non‐Resistant policy, but only conducted simulations for the behavior under the Resistant policy by assuming the initial value of *w* = *R* (=1). We did, however, calculated the RPEs generated in the model with reduced SR under the Non‐Resistant policy in the condition with *w* = 1, corresponding to the completion of learning under the Non‐Resistant policy, by using Equations (6), (9), and (11), resulting in that RPE = *γ^n^* upon initiation of behavior and RPE = 0 otherwise.

### 2.4 Slow update of the goal‐based reduced SR of states

In simulations with slow update of the goal‐based reduced SR itself, we updated the scalar feature of the state (i.e., *x*(*S*(*t*))) at every time step, except for the feature of the goal state (mentioned below), by using the TD error of the goal‐based reduced SR:

(12) $$\delta_{\text{feature}}=0+\gamma x\left(S\left(t+1\right)\right)‐x\left(S\left(t\right)\right).$$

Specifically, the scalar feature was updated as follows:

(13) $$x\left(S\left(t\right)\right)\to x\left(S\left(t\right)\right)+\alpha_{\text{feature}}\delta_{\text{feature}},$$

where *α*_feature_ is the learning rate for this update and was set to 0.05. As for the goal state, the TD error of the goal‐based reduced SR for the goal state should be theoretically 0 and thus no update was implemented.

### 2.5 Model with genuine SR of states

For comparison, we also considered a model with genuine SR of states. We assumed that each state *S_k_* is represented by *n* features *x_j_*(*S_k_*) (*j* = 1, …, *n*) indicating the time‐discounted future occupancy of *S_j_* under the Non‐Resistant policy:

(14) $$x_{j}\left(S_{k}\right)=\gamma^{j‐k}\left(j\geq k\right)\;\text{or}\;0\left(j<k\right),$$

and the (true) value function under the Resistant policy is approximated by a linear function of them:

(15) $$V_{\text{Resistant}}\left(S_{k}\right)\approx \Sigma_{j=1:n}\left\{w_{j}x_{j}\left(S_{k}\right)\right\}.$$

The coefficients *w_j_* (*j* = 1, …, *n*) are updated by using the (TD‐type) RPE:

(16) $$\delta_{\text{genuine}}=R\left(S\left(t\right)\right)+\gamma \Sigma_{j=1:n}\left\{w_{j}x_{j}\left(S\left(t+1\right)\right)\right\}‐\Sigma_{j=1:n}\left\{w_{j}x_{j}\left(S\left(t\right)\right)\right\},$$

where the middle term including *S*(*t* + 1) is dropped if *S*(*t*) is the goal state, according to the following rule:

(17) $$w_{j}\to w_{j}+\alpha_{\text{genuine}}x_{j}\left(S\left(t\right)\right)\delta_{\text{genuine}},$$

where *α*_genuine_ is the learning rate and was set to 0.5. The initial values of *w_j_* were set to 0 for *j* = 1, …, *n* − 1 and *R* (=1) for *j* = *n*, with which the approximate value function exactly matches the true value function under the Non‐Resistant policy.

### 2.6 Influence of the rigid reduced SR system on the system with individual action representation

For simulations of the influence of the rigid reduced SR system on the system with individual action representation, we assumed that the action values of “Go” and “No‐Go” in the system with individual action representation are updated by using a combination of the RPEs generated in the rigid reduced SR system and the RPEs of action values of either the Q‐learning‐type or the SARSA‐type. Specifically, we considered the action values

(18) $$Q\left({\text{Go}}_{S}\right)\;\text{and}\;Q\left(\text{No}‐{\text{Go}}_{S}\right),$$

for “Go” and “No‐Go” at state *S* (=*S*_1_, …, *S_n_*_−1_), respectively, and considered the RPE of Q‐learning type:

(19) $$\delta_{\text{QL}}=R\left(S\left(t\right)\right)+\gamma \max\left\{Q\left({\text{Go}}_{S(t)}\right),Q\left(\text{No}‐{\text{Go}}_{S(t)}\right)\right\}‐Q\left(A{\left(t‐1\right)}_{S(t‐1)}\right),$$

where “max” is the operation to take the maximum and *A*(*t* − 1)*_S_*_(_ *_t _*_− 1)_ is the action actually taken at state *S*(*t* − 1), or the RPE of SARSA‐type:

(20) $$\delta_{\text{SARSA}}=R\left(S\left(t\right)\right)+\gamma Q\left(A{\left(t\right)}_{S(t)}\right)‐Q\left(A{\left(t‐1\right)}_{S(t‐1)}\right),$$

where *A*(*t*)*_S_*_(_ *_t_*_)_ is the action actually chosen at state *S*(*t*). For both types, if *S*(*t*) is the goal state, the middle term is dropped, and if *t* is the initial time step within an episode, the last term is dropped. The value of the previous action, *Q*(*A*(*t* − 1)*_S_*_(_ *_t _*_− 1)_), was then assumed to be updated by a combination of either of these RPEs and the RPE generated in the system with rigid reduced SR (Equation 9):

$$\delta_{\text{RSR}}=R\left(S\left(t\right)\right)+\gamma wx\left(S\left(t+1\right)\right)‐wx\left(S\left(t\right)\right).$$

In particular, *Q*(*A*(*t* − 1)*_S_*_(_ *_t _*_− 1)_) was assumed to be updated as:

(21) $$Q\left(A{\left(t‐1\right)}_{S(t‐1)}\right)\to Q\left(A{\left(t‐1\right)}_{S(t‐1)}\right)+\alpha_{\text{combined}}\left((1‐\kappa )\delta_{\text{QL}}+\kappa \delta_{\text{RSR}}\right),$$

or

(22) $$Q\left(A{\left(t‐1\right)}_{S(t‐1)}\right)\to Q\left(A{\left(t‐1\right)}_{S(t‐1)}\right)+\alpha_{\text{combined}}\left((1‐\kappa )\delta_{\text{SARSA}}+\kappa \delta_{\text{RSR}}\right),$$

where *α*_combined_ is the learning rate, which was set to 0.5, and *κ* (0 ≤ *κ* ≤ 1) and 1 − *κ* represent the degrees of the effects of the RPEs generated in the system with rigid reduced SR and the system with individual action representation, respectively; *κ* was varied to be 0, 0.2, or 0.4. We assumed that these RPE calculations and updates are implemented in the circuits shown in Figure  7(a). Notably, as appeared in the above equations, we assumed that the RPEs containing the reward at *S*(*t*) are used to update the value of action taken at *t* − 1 (rather than at *t*). Initial values of the action values for “Go” were set to be the theoretical true values under the Non‐Resistant policy, specifically,

(23) $$Q\left({\text{Go}}_{\mathrm{Sk}}\right)=R\gamma^{n‐1‐k},$$

and initial values of the action values for “No‐Go” were set to be the values that were one time‐step discounted from the initial values for “Go” at the same states:

(24) $$Q\left({\text{No - Go}}_{\mathrm{Sk}}\right)=R\gamma^{n‐k}.$$

The initial value of *w* was set to *R* (=1), corresponding to the completion of learning under the Non‐Resistant policy.

For comparison, we also conducted simulations of a model that was the same as the one described above except that the rigid reduced SR system, generating *δ*_RSR_, was replaced with the simple RL model with punctate (individual) state representation, generating *δ*_simple_ (Equation 2).

### 2.7 Execution of simulations

The agent's behavior under the Non‐Resistant policy is deterministic, and so we made theoretical calculations or conducted a single simulation (without using pseudorandom number) as for the results for the Non‐Resistant policy. Regarding the results for the Resistant policy, in order to examine average behavior of the model across simulations using pseudorandom numbers, simulations were conducted 100 times for each condition. Among the 100 simulations, there were likely to be simulations, where “No‐Go” choice was not taken at some state(s) at some episode(s). Such simulations, different from case to case, were not included in the calculations of the average and standard deviation of RPEs across simulations. There were also likely to be simulations, where “No‐Go” choice was taken more than once at some state(s) at some episode(s). In such cases, generated RPEs were first averaged within an episode, and that value (i.e., a single value for each simulation) was used for the calculations of the average and standard deviation of RPEs across simulations. Simulations and figure drawing were conducted by using Python and R, respectively.

### 2.8 Data availability statement

Program codes for generating all the data presented in the figures are available in the GitHub (https://github.com/Kshimod/Reduced_SR_RL).

## 3 RESULTS

### 3.1 Modeling nonaddicted versus addicted cases by models with simple RL versus rigid goal‐based reduced SR

We modeled a person's series of actions to obtain a certain reward, such as alcohol, nicotine, or nonsubstance such as betting ticket, gaming, or social interaction, by a series of modeled person's actions on a sequence of states from the start state to the goal state, where the reward is given (Figure 1[a]). At each state except for the goal state, the person can take either of two actions, “Go”: proceed to the next state, and “No‐Go”: stay at the same state (as considered in our previous work, Kato & Morita, 2016, in a different context). We considered a case that the person has long been regularly taking behavior to obtain the reward without resisting temptation. In the model, it corresponds to that the person has long experienced transitions towards the rewarded goal according to a policy that takes only “Go” at any state, which we refer to as the Non‐Resistant policy (Figure 1[a]‐b). We assumed that through such long‐standing experiences of behavior according to the Non‐Resistant policy, the person has potentially established a particular state representation, where each state is represented by the discounted future occupancy of the final successor state, namely, the rewarded goal state, under that policy (formulae and equations are described in Section 2).

This representation, which we will refer to as the goal‐based reduced SR (Figure 1[b]), can be said to be a dimension‐reduced version of SR; in the genuine SR (Dayan, 1993; Gershman, 2018; Russek et al., 2017), every state is represented by a vector of expected cumulative discounted future state occupancies for all the states, whereas in the above goal‐based reduced SR, every state is represented by the discounted future occupancy of only the goal state. Because the genuine SR requires the number of features equal to the number of states, dimension reduction has been considered (c.f., Barreto et al., 2016; Gardner et al., 2018; Gehring, 2015). Given the general suggestion of dimension reduction in state representations in the brain (Gershman & Niv, 2010; Niv, 2019), it would be conceivable that the brain adopts dimension‐reduced versions of SR, such as the goal‐based reduced SR assumed above. Notably, the state value function under the Non‐Resistant policy in the assumed state and reward structure (Figure 1[a]‐a) can be precisely represented as a linear function of the scalar feature of the goal‐based reduced SR (Equation 8 in Section 2). Moreover, this representation inherits the sensitivity to changes in the reward value at the goal from the genuine SR, and thus, the agent (person) having acquired this representation remains to be goal‐directed in terms of sensitivity to changes in the goal value. It would thus be conceivable that such a goal‐based reduced SR can be acquired through long‐standing reward‐obtaining behavior.

We propose that such a goal‐based reduced SR under the Non‐Resistant policy can be established so rigidly that it cannot be updated, depending on the property of reward, duration and frequency of nonresistant reward‐obtaining, and individuals. We tentatively refer to the case with establishment of such a rigid reduced SR as the addicted case, and other case as the nonaddicted case; later at the beginning of Section 4, we will discuss that the addicted case so defined as above is potentially in line with several defining characteristics of addiction. For the nonaddicted case, we assumed that each state is represented individually (or in the “punctate” manner using the terminology in Russek et al., 2017) as in conventional simple RL models; we will also show other possibility for the nonaddicted case later (in the fourth section).

### 3.2 Behavior of the simple RL model, simulating the nonaddicted case

**FIGURE 2:**
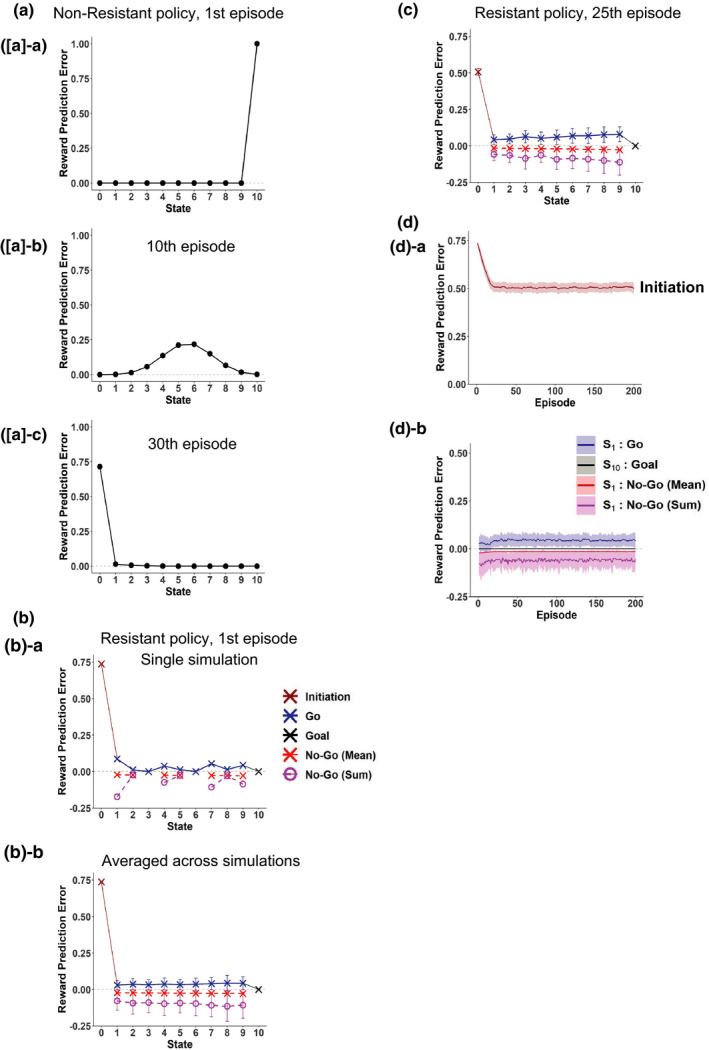
RPEs generated in the simple RL model with individual (punctate) state representation, simulating the nonaddicted case. (a) RPEs generated in the first episode ([a]‐a), 10th episode ([a]‐b), and 30th episode ([a]‐c) under the Non‐Resistant policy, starting from the initial condition where the value of every state was 0. RPE upon initiation of behavior is also shown in the leftmost *S*_0_ position. (b)‐a A single‐simulation example of RPEs generated in the first episode under the Resistant policy, starting from the initial condition corresponding to the completion of learning under the Non‐Resistant policy. The blue crosses indicate RPEs generated upon “Go” decisions, whereas the red crosses indicate the means of RPEs generated upon “No‐Go” decisions, and the brown and black crosses indicate RPEs generated upon initiation of behavior and at the goal state, respectively. The magenta circles indicate the summation of RPEs generated upon “No‐Go” decisions at the same states. (b)‐b Mean RPEs generated in the first episode under the Resistant policy. The error bars indicate the average ± *SD* across simulations; this is also applied to the following figures unless otherwise mentioned. (c) Mean RPEs generated in the 25th episode under the Resistant policy. (d) The changes of RPEs over episodes under the Resistant policy. The shading indicates the average ± *SD* across simulations; this is also applied to the following figures. (d)‐a RPEs generated upon initiation of behavior. (d)‐b RPEs generated upon “Go” decisions (blue) and “No‐Go” decisions (mean (red) and summation (magenta) per episode) at the start state, and RPE generated at the goal state (black)

Here, we first present the nonaddicted case simulated by a conventional simple RL model with individual (punctate) state representation, and as compared to it, we will show the addicted case simulated by a model with the rigid goal‐based reduced SR in the next section. We simulated that the person initially learned the values of each state leading to the goal state, where a reward was obtained, under the Non‐Resistant policy by setting the initial value for the state value of each state to 0. Figure 2(a) shows the RPEs generated at each state in the first, 10th, and 30th episode, also showing the RPE upon initiation of behavior (in the leftmost *S*_0_ position), which was assumed to be the learned state value of *S*_1_ multiplied by the time discount factor. As shown in the figure, in the first episode, a large positive RPE was generated at the goal state, while no RPE was generated elsewhere. By contrast, in the 30th episode, a large positive RPE was generated upon initiation of behavior, while RPE at the goal faded away. This disappearance of RPE for repeatedly experienced reward is a hallmark of the conventional temporal difference (TD) RL model (Sutton & Barto, 2018), and this pattern resembles the pattern of DA response in the process of learning the value of (nonaddictive) reward (Montague et al., 1996; Schultz et al., 1997).

Let us then consider a situation where the person decides to attempt cessation of the series of reward‐obtaining behavior. We assumed that the person starts to take a new policy, referred to as the Resistant policy, in which not only “Go” but also “No‐Go” action is chosen with a certain probability, *P*_No‐Go_, at each state preceding the goal (Figure 1(a)‐b). We simulated the person's behavior under the Resistant policy with *P*_No‐Go_ = 0.75 starting from the initial condition that corresponds to the completion of learning under the Non‐Resistant policy. Figure 2(b)‐a shows a single simulation example of RPEs generated in the first episode. In this episode, the person chose “No‐Go” once at *S*_2_, *S*_5_, and *S*_8_, three times at *S*_4_ and *S*_9_, four times at *S*_7_, eight times at *S*_1_, and never at *S*_3_ and *S*_6_. The blue crosses indicate RPEs generated upon “Go” decisions, whereas the red crosses indicate the means of RPEs generated upon “No‐Go” decisions, and the brown and black crosses indicate RPEs generated upon initiation of behavior and at the goal state, respectively. The magenta circles indicate the summation of RPEs generated upon “No‐Go” decisions at the same states. As shown in the figure, a large positive RPE was generated upon initiation of behavior, and small positive and negative RPEs were generated when the person chose “Go” and “No‐Go” at each state, respectively, whereas no PRE was generated when the person eventually reached the rewarded goal state. Figure 2(b)‐b shows the mean and standard deviation across simulations. The same features as observed in the example simulation are observed. Figure 2(c) shows the RPEs generated at the 25th episode, averaged across simulations. Compared to the case of the first episode, the magnitude of RPE upon initiation of behavior was reduced, and this is considered to reflect that more time steps were needed for goal reaching under the Resistant policy than under the Non‐Resistant policy and so more temporal discounting was imposed. On the other hand, similarly to the case of the first episode, small positive and negative RPEs were generated upon “Go” and “No‐Go” choices, respectively, and no RPE was generated upon goal reaching. These patterns were largely preserved after the 25th episode, as shown in Figure 2(d).

### 3.3 Behavior of the model with rigid reduced SR, simulating the addicted case

**FIGURE 3:**
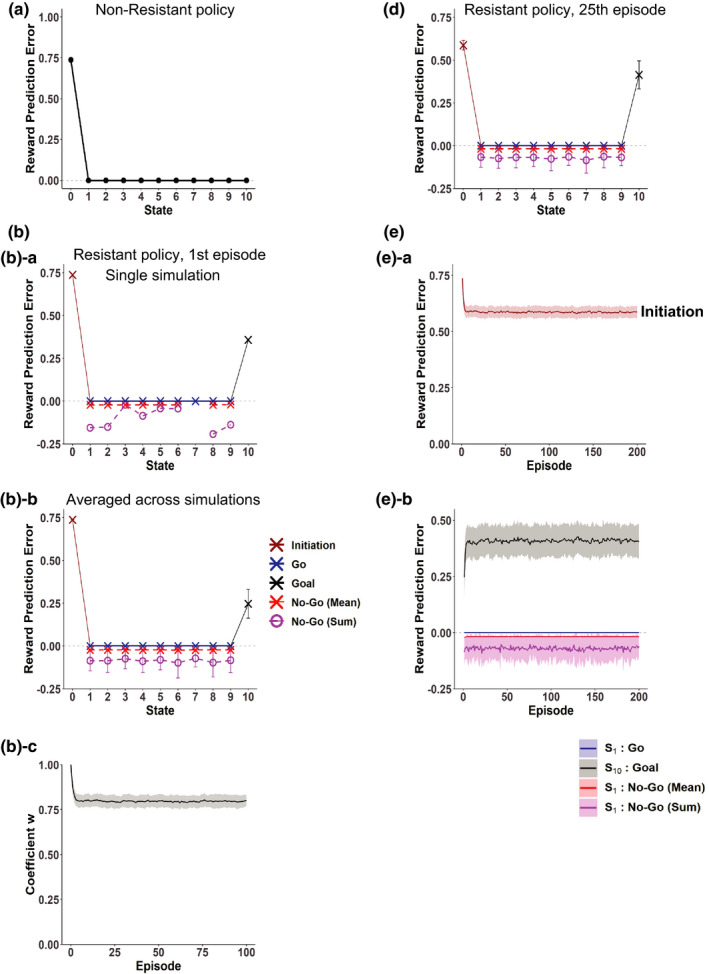
RPEs generated in the model with rigid goal‐based reduced SR established under the Non‐Resistant policy, simulating the addicted case. (a) RPEs generated under the Non‐Resistant policy, in the condition where the coefficient of the approximate value function (*w*) was 1, corresponding to the completion of learning under the Non‐Resistant policy. (b)‐a A single‐simulation example of RPEs generated in the first episode under the Resistant policy, starting from the initial condition corresponding to the completion of learning under the Non‐Resistant policy. (b)‐b Mean RPEs generated in the first episode under the Resistant policy. (c) Over‐episode change of the coefficient *w* of the approximate value function at the end of each episode under the Resistant policy; the assumed initial value (*w* = 1) is also plotted at episode = 0 with *SD* = 0. (d) Mean RPEs generated in the 25th episode under the Resistant policy. (e) The changes of RPEs over episodes under the Resistant policy. (e)‐a RPEs generated upon initiation of behavior. (e)‐b RPEs generated at the start state and the goal state

We now present the addicted case simulated by the model with rigid goal‐based reduced SR. We assumed that the goal‐based reduced SR of states had been formed through long‐standing behavior under the Non‐Resistant policy, although we did not model the formation process itself. We thus considered approximation of the state value function by a linear function of the features of the reduced SR, that is, the discounted future occupancy of the goal state. Figure 3(a) shows the RPEs generated under the Non‐Resistant policy, in the condition where the coefficient of the approximate value function (*w*) was 1, corresponding to the completion of learning under the Non‐Resistant policy. As shown in the figure, a large positive RPE was generated upon initiation of behavior, and no RPE was generated elsewhere. This is very similar to the nonaddicted case modeled by the simple RL model (Figure 2[a]‐c).

Next, we present the results for the Resistant policy. Similarly to the case of the simple RL model in the previous section, we simulated the person's behavior under the Resistant policy with *P*_No‐Go_ = 0.75 starting from the initial condition that corresponds to the completion of learning under the Non‐Resistant policy (i.e., *w* = 1). Figure 3(b)‐a shows a single simulation example of RPEs generated in the first episode. In this episode, the person chose “No‐Go” once at *S*_3_, twice at *S*_5_ and *S*_6_, four times at *S*_4_, seven times at *S*_1_, *S*_2_, and *S*_9_, nine times at *S*_8_, and never at *S*_7_. As shown in the figure, a large positive RPE was generated upon initiation of behavior, and small negative RPEs were generated when the person chose “No‐Go”, whereas theoretically no RPE is generated upon choosing “Go” (though tiny numerical errors existed [the same applies throughout]). Then, when the person eventually reached the rewarded goal state, a relatively large positive RPE was generated, different from the nonaddicted case modeled by the simple RL model shown in the previous section. Figure 3(b)‐b shows the mean and standard deviation across simulations. The same features as observed in the example simulation are observed.

Figure 3(c) shows the over‐episode change of the coefficient *w* of the approximate value function at the end of each episode, averaged across simulations. As shown in the figure, *w* decreases from its initial value (=1) and becomes (almost) stationary, meaning that the negative and positive RPE‐based updates become overall balanced. We examined RPEs after the coefficient *w* becomes nearly stationary, in particular, in the 25th episode. Figure 3(d) shows the results averaged across simulations. Compared to the case of the first episode, the magnitude of RPE upon initiation of behavior was reduced. The reduction looks, however, less prominent than the nonaddicted case modeled by the simple RL. This is considered to be because even though the person actually needs much more time steps for goal‐reaching according to the Resistant policy, a sort of memory of nonresistant fast goal‐reaching is “imprinted on” the established reduced SR under the Non‐Resistant policy and affects the estimation of state values. At the states preceding the goal, small negative RPEs were generated upon “No‐Go” decisions, whereas theoretically no RPE is generated upon “Go” decisions, similarly to the case of the first episode. Then, when the person eventually reached the goal state, a large positive RPE, whose mean magnitude was larger than that in the first episode, was generated. This is, again, clearly different from the nonaddicted case modeled by the simple RL.

We also examined how the amplitudes of RPEs change over episodes, and found that after a few initial episodes, the amplitudes, averaged across simulations, become nearly stationary (Figure 3[e]). In particular, the large positive RPE generated at the rewarded goal sustains after many repetitions. This is quite different from the conventional diminishing RPE for repetitive (nonaddictive) reward, and somewhat similar to the hypothesized fictitious RPE caused by addictive drug‐induced DA (Keiflin & Janak, 2015; Redish, 2004). But importantly, our model does not assume any direct modulation of the DA system by substance, and thus, such a sustained large positive RPE for repetitive reward in the addicted case only originates from the formation of the goal‐based reduced SR under the Non‐Resistant policy and its rigidity. More specifically, the difference between the nonaddicted case with simple RL and the addicted case with rigid reduced SR is considered to reflect different characteristics of updates done with the different ways of state representation. Specifically, in the case with the goal‐based reduced SR, only the coefficient of approximate value function was updated and the state representation established under the Non‐Resistant policy was (assumed to be) unchanged, resulting in sustained mismatch between the true and approximate value functions. In contrast, in the case of the simple RL with individual (punctate) state representation, the value of each state was directly updated so that there is no such sustained mismatch. For both the cases of reduced SR and simple RL, we examined the cases with different parameters, and found that basic features of the patterns of sustained RPEs were largely preserved (see the Supporting Information).

**FIGURE 4:**
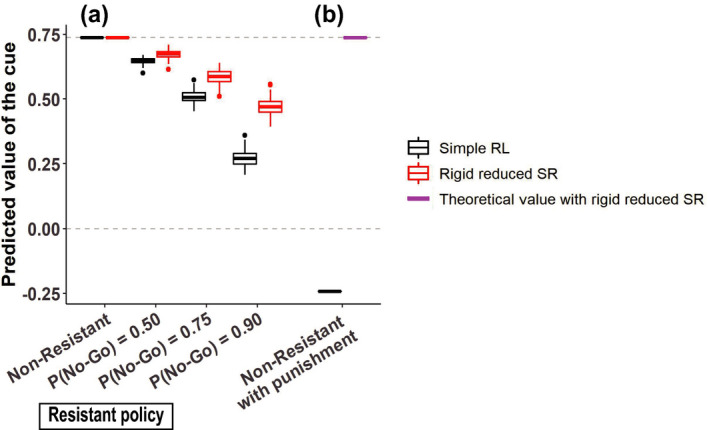
Decrease in the predicted value of the cue for behavior leading to reward by resistance to temptation, and the effects of punishment. (a) The predicted value of the cue for behavior leading to size 1 reward at the completion of learning under the Non‐Resistant policy (leftmost) or at the 25th episode under the Resistant policy with *P*_No‐Go_ = 0.5, 0.75, or 0.9 starting from the initial condition corresponding to the completion of learning under the Non‐Resistant policy. *Black symbols*: the nonaddicted case modeled with simple RL. *Red symbols*: the addicted case modeled with rigid reduced SR. (b) Effects of subsequent introduction of punishment at the state following the goal state. The black symbol indicates the predicted value of the cue at the 25th episode after the introduction of size 2 punishment under the Non‐Resistant policy in the nonaddicted case modeled with simple RL, starting from the initial condition corresponding to the completion of learning without punishment. In the addicted case, the predicted value of the cue is theoretically considered to be unchanged from the value without punishment, as indicated by the magenta symbol, because of the reason described in Section 3

As mentioned above, the addicted case modeled with rigid reduced SR and the nonaddicted case modeled with simple RL differed also in the degree of over‐episode reduction of the RPE upon initiation of behavior under the Resistant policy. Let us consider a situation in which there is a certain cue for behavior leading to reward. The predicted value of such a cue is considered to be equal to the RPE generated upon initiation of behavior. We examined how the predicted value of the cue changed when the person took the Resistant policy with different degrees of strictness (*P*_No‐Go_ = 0.5, 0.75, or 0.9), comparing the nonaddicted and addicted cases. Figure 4(a) shows the results. As shown in the figure, in both cases, continued resistance resulted in a decrease in the predicted value of the cue, and the degree of the decrease depended on the strictness of the resistance, but the decrease was less prominent in the addicted case (red symbols) than in the nonaddicted case (black symbols). This is considered to contribute to making cessation of reward‐obtaining behavior more difficult in the addicted case than in the nonaddicted case.

The addicted case modeled with rigid reduced SR and the nonaddicted case modeled with simple RL are expected to further differ in the responsiveness to subsequent introduction of punishment at the state following the rewarded goal state. Specifically, in the nonaddicted case modeled with simple RL, such subsequent introduction of punishment causes a reduction of the learned value of each state and thereby a reduction of the predicted value of the cue for behavior, and if the punishment is large enough, the cue value becomes negative (Figure 4(b), black). By contrast, in the addicted case modeled with rigid reduced SR, given that no backward transition from the state following the goal state to the goal state has been experienced, the scalar feature (discounted future occupancy of the goal state) of the state following the goal state can be considered to be 0. Then, even though the punishment causes negative RPE at the state following the goal state, it does not cause an update of the coefficient of the approximate value function (because *x*(*S*(*t*)) in Equation 10 in Section 2 is 0) and thus does not reduce the learned cue value (Figure 4(b), magenta), unless the state representation itself will change.

### 3.4 Cases where goal‐based reduced SR is not rigid but can be updated or genuine SR is used

**FIGURE 5:**
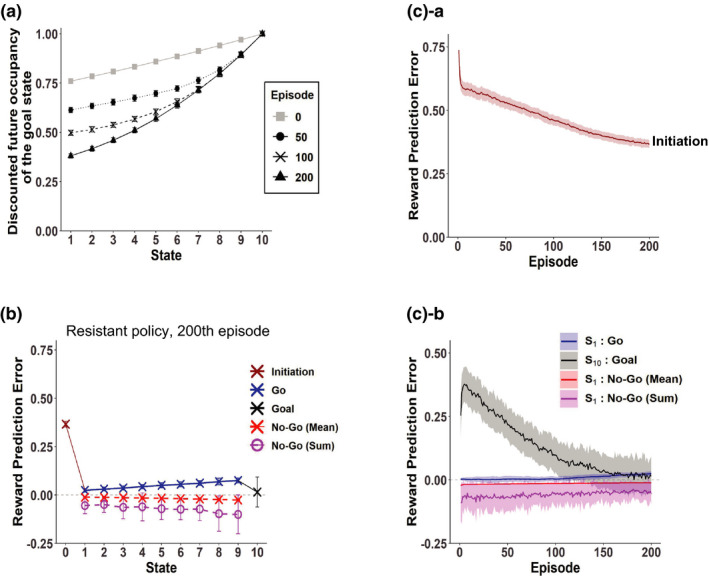
RPEs generated under the Resistant policy in the case where the goal‐based reduced SR established under the Non‐Resistant policy itself slowly changed and approached the goal‐based reduced SR under the Resistant policy. (a) Scalar feature of each state (i.e., *x*(*S_k_*)) after 50, 100, and 200 episodes (black dotted, dashed, and solid lines, respectively), in comparison to the original ones (gray line) that are the same as those shown in Figure 1(b)‐b. (b) Mean RPEs generated in the 200th episode. (c) The changes of RPEs over episodes under the Resistant policy

In the previous section, we considered rigid reduced SR that cannot be updated after the policy has been changed. Here we consider the case where goal‐based reduced SR is once established under the Non‐Resistant policy but it can be slowly updated after the policy is changed to the Resistant policy through TD learning of state representation itself (Gardner et al., 2018; Gershman et al., 2012). Figure 5(a) shows the scalar feature of each state (i.e., discounted future occupancy of the goal state) after 50, 100, and 200 episodes under the Resistant policy (black dotted, dashed, and solid lines, respectively), averaged across simulations, in comparison to the original ones established under the Non‐Resistant policy (gray line). As shown in the figure, the curve became steeper as episodes proceeded. This is considered to reflect that longer time is required, on average, for goal reaching under the Resistant policy than under the Non‐Resistant policy and thus the expected discounted future occupancy of the goal state should be smaller for the Resistant policy. Figure 5(b) shows the RPEs generated in the 200th episode, averaged across simulations, and Figure 5(c) shows the over‐episode changes in the RPEs. As shown in these figures, a large positive RPE was initially generated upon goal reaching but it gradually decreased, while positive RPEs with smaller amplitudes gradually appeared upon “Go” decisions in the states other than the goal, and so the pattern of RPEs gradually approached that of the simple RL model in these regards. Notably, the RPE upon initiation of behavior became even smaller than the case of simple RL (compare Figures 2[d]‐a and 5[c]‐a), presumably reflecting that whereas infrequent fast reaching may greatly raise the RPE in the simple RL, the average slow reaching would be imprinted on the slowly updated reduced SR, resulting in the smaller RPE. These results indicate that even if goal‐based reduced SR under the Non‐Resistant policy is once established, if it is not so rigid that it can be updated albeit slowly, cessation of reward‐obtaining would eventually become less difficult if the person does not give up resisting temptation.

**FIGURE 6:**
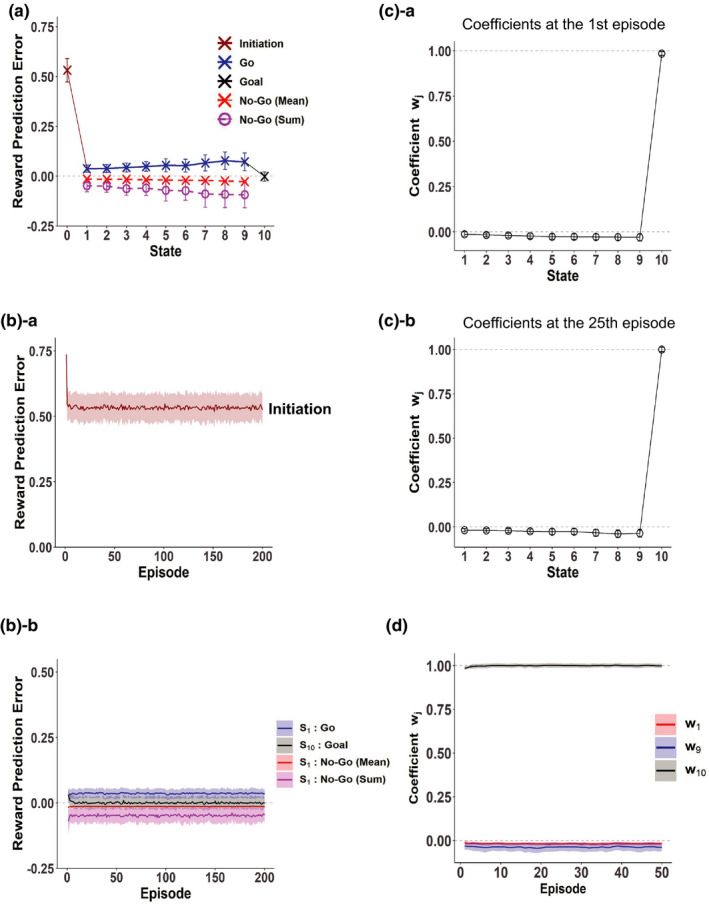
RPEs generated under the Resistant policy in the model with genuine SR. (a) Mean RPEs generated in the 25th episode. (b) The changes of RPEs over episodes under the Resistant policy. (c) Coefficients *w_j_* of the approximate value function after the first episode ([c]‐a) and 25th episode ([c]‐b). (d) Over‐episode changes of the coefficients *w_j_* for the features corresponding to the start state (red line), the state preceding the goal (*S*_9_) (blue line), and the goal state (black line)

We also considered a case where the states are represented by the genuine SR, rather than the reduced SR. Figure 6(a) shows the RPEs generated in the 25th episode, and Figure 6(b) shows the over‐episode changes in the RPEs. As shown in the figures, the patterns of RPEs are similar to those in the case of the simple RL model with individual (punctate) state representation (Figure  2[b], [c]) and differ from those in the case of the model with the rigid reduced SR. Therefore, cessation of reward‐obtaining is considered to be not very difficult in this case, or in other words, this case is also considered to be a nonaddicted case. Figure 6(c) shows the coefficients *w_j_* of the approximate value function after the 1st episode (Figure 6(c)‐a) and 25th episode (Figure 6(c)‐b), and Figure 6(d) shows the over‐episode changes of the coefficients for the features corresponding to the start state (red line), the state preceding the goal (*S*_9_) (blue line), and the goal state (black line). As shown in these figures, the coefficients for the features corresponding to the states preceding the goal became negative. It is considered that because of these negative coefficients, the true value function under the Resistant policy could be well approximated even by a linear function of the features (discounted occupancies) under the Non‐Resistant policy.

### 3.5 Influence of the rigid reduced SR system on the system with individual action representation

**FIGURE 7:**
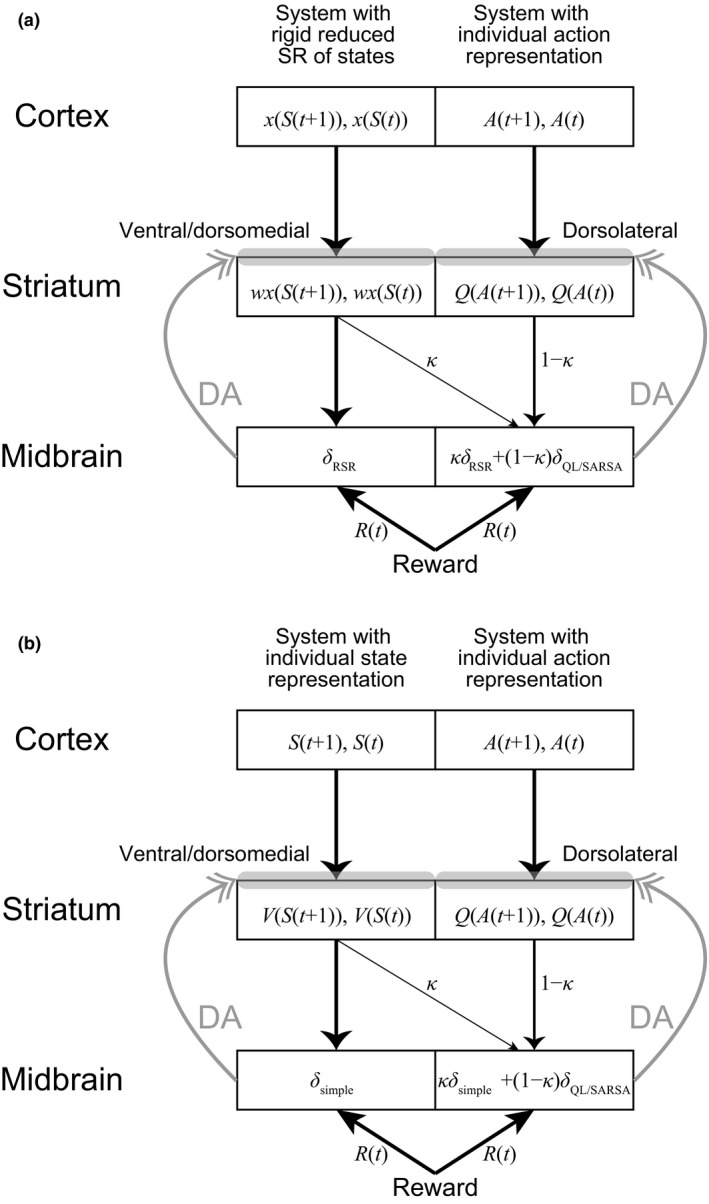
Schematic illustration of the spiral striatum‐midbrain circuit and the hypothesized influence of the RPE generated in the circuit including the ventral/dorsomedial striatum on the circuit including the dorsolateral striatum. (a) The case where the ventral/dorsomedial circuit hosts the system with goal‐based reduced SR of states whereas the dorsolateral circuit hosts the system with individual action representation. In the ventral/dorsomedial circuit (left), the cortex represents the features (discounted future occupancies of the goal state) of the upcoming and previous states and the striatum represents their approximate state values obtained by the linear function of the features with the coefficient *w*. In the dorsolateral circuit (right), the cortex represents the upcoming and previous actions and the striatum represents their action values. The oblique line indicates the influence through the spiral striatum‐midbrain projections, and *κ* (0 ≤ *κ* ≤ 1) and 1 − *κ* represent the degrees of the effects of the RPEs generated in the ventral/dorsomedial circuit and dorsolateral circuit, respectively, on the dorsolateral circuit. (b) The case where the ventral/dorsomedial circuit hosts the system with individual state representation while the dorsolateral circuit hosts the system with individual action representation. In the ventral/dorsomedial circuit (left), the cortex represents the upcoming and previous states and the striatum represents their state values

As mentioned in Section 1, it is suggested that there exist multiple value learning systems in the brain, with the system employing SR residing in the prefrontal/hippocampus‐dorsomedial/ventral striatum circuits. Another system adopting individual (punctate) representation might locate in the circuits including dorsolateral striatum. Moreover, there are anatomical suggestions of ventral‐to‐dorsal spiral influences in the striatum‐midbrain system (Haber et al., 2000; Joel & Weiner, 2000), and theoretical proposals that such a spiral circuit implements heterarchical RL (Haruno & Kawato, 2006) and that the bias of RPE due to drug‐induced DA accumulates through the spiral circuit and causes undesired compulsive drug taking in long‐term addicts (Keramati & Gutkin, 2013). Inspired by these, we also examined a case with multiple representation/learning systems. Specifically, we assumed that the prefrontal/hippocampus‐dorsomedial/ventral striatum circuits host the goal‐based reduced SR of states (rather than the genuine SR) whereas the circuits including dorsolateral striatum adopt an individual (punctate) representation of each action, that is, “Go” or “No‐Go”. This latter assumption was made based on the suggestions that the dorsal/dorsolateral striatum is involved in value learning with actions (O'Doherty et al., 2004; Takahashi et al., 2008). We then assumed that the information of the RPEs generated in the system with rigid goal‐based reduced SR of states flows into the system with punctate (i.e., individual) action representation through the spiral circuit (Figure 7(a)). Critically, different from the abovementioned previous model (Keramati & Gutkin, 2013), which assumed that the value of the upcoming state but not of the previous state in the ventral circuit flows into the dorsal circuit, we assumed that the information of the values of both upcoming and previous states used for (TD‐type) RPE calculation originates from the striatum, and effectively the entire RPE in the ventral circuit flows into the dorsal circuit (in this regard, somewhat similar assumption was made in [Takahashi et al., 2008]). If both the upcoming and previous values are sent via the direct striatum‐midbrain connections, for example, through the matrix and patch/striosomal neurons as referred to in Morita et al. (2012), the suggested spiral connections could also convey both information, though it needs to be verified. If either value (or both) is sent to the midbrain via the indirect pathway through the globus pallidus or ventral pallidum, as proposed in (Doya, 2000; Houk et al., 1995; Morita & Kawaguchi, 2019; Morita et al., 2012), our assumption requires spiral connectivity for both direct and indirect pathways, which also needs to be validated. We also noticed that a recent study specifically suggested a function of the hierarchical cortico‐basal ganglia circuits in habit learning (Baladron & Hamker, 2020), but our model considers a different mechanism.

Regarding the system with individual action representation, we assumed that “Go” and “No‐Go” at each state other than the goal state are represented in a punctate manner (i.e., individually) and their values (i.e., action values) are updated by using a combination of the RPEs generated in the rigid reduced SR system and the RPEs of action values. As for the latter, we considered two types: the Q‐learning‐type and the SARSA‐type, both of which have been suggested to be represented by DA (Morris et al., 2006; Roesch et al., 2007). We conducted simulations of behavior under the Resistant policy (*P*_No‐Go_ = 0.75) starting from the initial condition corresponding to the completion of learning under the Non‐Resistant policy as for the “Go” values and the coefficient of the approximate value function (initial values of the “No‐Go” values were also set in a reasonable way, as described in Section 2), with the degrees of the effects of the RPE in the rigid reduced SR system and the RPE in the system with individual action representation varied (*κ* and 1 − *κ*, respectively, in Equations (20) and (21) in Section 2).

**FIGURE 8:**
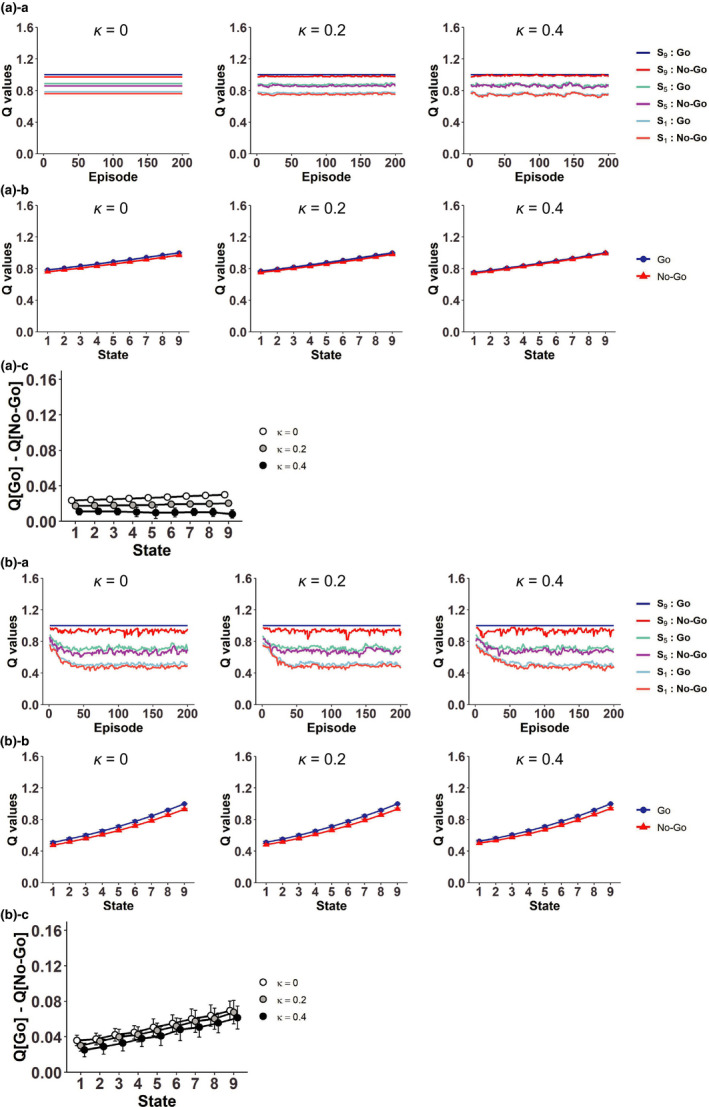
Influence of the RPEs generated in the system with goal‐based reduced SR of states to the system with individual action representation. (a) Results with the Q‐learning‐type RPE of action values. ([a]‐a) Examples of over‐episode changes of the values of “Go” and “No‐Go” at the start state, the middle (5th) state, and the pre‐goal (9‐th) state in the case with different degrees of the relative effect of the RPE generated in the system with goal‐based reduced SR of states (*κ* = 0 (left panels), 0.2 (middle panels), and 0.4 (right panels)) in single simulations. ([a]‐b) The values of “Go” (blue lines) and “No‐Go” (red lines) at each state in the case with *κ* = 0 (left panels), 0.2 (middle panels), and 0.4 (right panels), averaged across the 41st to 60th episodes and also across simulations. The error bars indicate ± *SD* across simulations. ([a]‐c) The differences of the values of “Go” and “No‐Go” at each state (*Q*(Go*_Sk_*) − *Q*(No‐Go*_Sk_*)) in the case with *κ* = 0, 0.2, and 0.4, averaged across the 41st to 60th episodes and also across simulations. The error bars indicate ± *SD* across simulations. (b) Results with the SARSA‐type RPE of action values. Configurations are the same as those in (a)

Figure 8(a)‐a shows examples of across‐episode changes of the values of “Go” and “No‐Go” at the start state, the middle (5th) state, and the pregoal (9th) state in the cases in single simulations with the Q‐learning‐type RPE. When there was no influence of the rigid reduced SR system (*κ* = 0), all the action values look unchanged from their initial values, as theoretically expected. As the relative influence of the rigid reduced SR system increased (*κ* = 0.2 and *κ* = 0.4), the values of “Go” and “No‐Go” at the start and middle states initially decreased, but the values of “Go” and “No‐Go” at the pregoal state increased, and eventually the action values at all these three states became larger than the values in the case without the influence of the rigid reduced SR system. The initial decreases of action values at the start and middle states are considered to be because of the negative RPEs upon “No‐Go” choices in the rigid reduced SR system, whereas the eventual increases of action values are considered to come from the large positive RPE upon goal‐reaching in the rigid reduced SR system.

Figure 8(b)‐a shows examples of the “Go” and “No‐Go” values in single simulations with the SARSA‐type RPE. When there was no influence of the rigid reduced SR system (*κ* = 0), the values of actions except for “Go” at the pregoal state generally decreased from their initial values. This is reasonable, because the on‐policy values of these actions under the Resistant policy should be smaller than the values under the Non‐Resistant policy due to extra time steps required for goal reaching. As the relative influence of the rigid reduced SR system increased (*κ* = 0.2 and *κ* = 0.4), the values of “Go” and “No‐Go” at the pre‐goal state increased, presumably due to the large positive RPE upon goal reaching in the rigid reduced SR system, while the effects on the action values at the start and middle states appear to be more mixed.

Figure 8(a)‐b shows the values of “Go” and “No‐Go” at each state, and Figure 8(a)‐c shows their differences (*Q*(Go*_Sk_*) − *Q*(No‐Go*_Sk_*)), averaged across the 41st to 60th episodes and also across simulations, with the Q‐learning‐type RPE. Figure 8(b)‐b,c shows the results with the SARSA‐type RPE. As shown in these figures, in both cases with the different types of RPE of action values, the values of “Go” were on average larger than the values of “No‐Go”, and the value difference on average increased as the relative influence of the rigid reduced SR system increased (*κ* = 0.2 and *κ* = 0.4), although there were large variations in the case of the SARSA‐type RPE. Therefore, if the “Go” and “No‐Go” values were assumed to affect the agent's choice propensity, which was in reality predetermined to be a fixed probability (*P*_No‐Go_ = 0.75) in our model as described above, the RPE information flowing from the rigid reduced SR system to the system with individual action representation through the spiral circuit could potentially enhance deterioration of the resistance to temptation. This result is intuitively understandable because, from the standpoint of the system with individual action representation, the incoming positive RPE from the rigid reduced SR system upon goal reaching would act as an extra reward.

**FIGURE 9:**
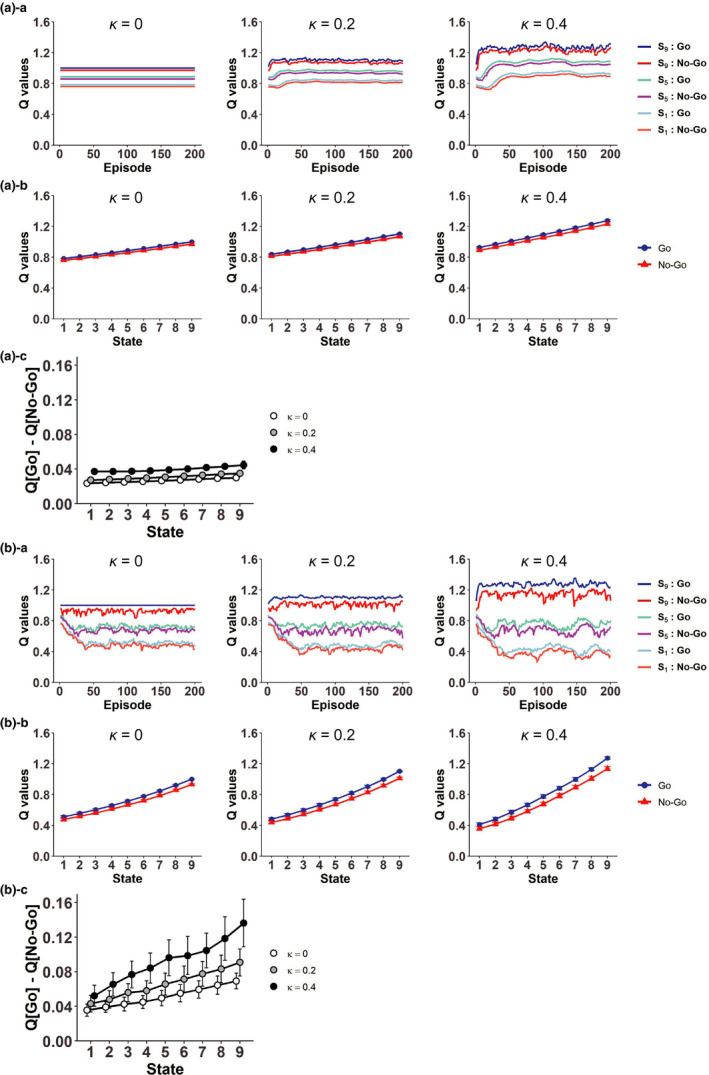
Influence of the RPEs generated in the system with individual state representation to the system with individual action representation. Configurations are the same as those in Figure 8, except that *κ* in this figure represents the degree of the effect of the RPE generated in the system with individual state representation. (a) Results with the Q‐learning‐type RPE of action values. (b) Results with the SARSA‐type RPE of action values

For comparison, we also examined the case where the information of the RPEs generated in the simple RL model with individual *state* representation, rather than the system with the rigid reduced SR of states, flows into the system with individual *action* representation (Figure 7(b)). Figure 9 shows the results. Different from the case with the RPE influence from the rigid reduced SR system, the RPE influence from the simple RL model did not increase but rather decreased the differences between the “Go” and “No‐Go” values, in both cases with Q‐leaning type (Figure 9(a)‐c) or SARSA‐type (Figure 9(b)‐c) RPE of action values. As shown before (Figure 2), in the simple RL model with individual state representation, negative and positive RPEs are generated upon “No‐Go” and “Go” choices, respectively. However, through the influence to the system with individual action representation, these RPEs are used for updating the value of the *previous* action, which is either “No‐Go” at the same state or “Go” at the preceding state (except when the agent is at the start state). Therefore, the negative and positive RPEs upon “No‐Go” and “Go” choices in the simple RL model do not directly affect the values of chosen actions themselves. These results indicate that the spiraling RPE influence from the rigid reduced SR system, but not from the simple RL model, could potentially enhance deterioration of the resistance to temptation.

## 4 DISCUSSION

We assumed that long‐standing behavior to obtain a certain reward without resistance to temptation can lead to a formation of rigid goal‐based reduced SR. Then we have shown that if it is formed, (1) while no RPE is generated at the goal as far as the person does not resist temptation, a sustained large positive RPE is generated upon goal reaching once the person starts to resist, (2) resistance‐dependent decrease in the predicted value of the cue becomes less prominent, (3) subsequent introduction of punishment at the state following the goal does not reduce the predicted value of the cue, and (4) influence of RPEs on the system with individual action representation through the spiral striatum‐midbrain circuit could potentially enhance the propensity of nonresistant choice. Defining characteristics of addiction include (i) craving or urge, (ii) inability to manage to stop, (iii) occurrence of relapse, and (iv) continuation despite loss or problem. The above (1)–(4) are considered to be potentially related to these characteristics, in particular (1) and (4) to (i)–(iii), (2) to (i) and (iii), and (3) to (iv). Therefore, we propose that formation of rigid reduced SR is a potential mechanism for addiction, common to substance and nonsubstance reward.

### 4.1 Further possibilities about the effects of the generated RPEs on behavior

As shown in Section 3, the large positive RPE at the goal generated in the rigid reduced SR system could act as an extra reward for the system with individual action representation. In the worse case, we speculate that it could potentially even act as fictitious RPEs that cannot be fully canceled out by predictions within the action representation system and thereby causes unbounded value increase and compulsion, similarly to what has been suggested for drug‐induced DA (Redish, 2004). The anatomically suggested ventral‐to‐dorsal spiral influences (Haber et al., 2000; Joel & Weiner, 2000) more precisely refer to the projections of more ventral parts of striatum to more dorsal parts of midbrain. Therefore, if every DA neuron in the dorsal parts of midbrain receives value information from both the ventral and dorsal parts of striatum with a fixed ratio as assumed in Figure 7(a), positive RPEs generated in the reduced SR system can be canceled out by negative RPEs of action values at the level of inputs to the DA neuron. However, if there exist some DA neurons in the dorsal parts of midbrain that receive value information only from the ventral parts of striatum, such a cancelation cannot occur at the level of inputs. Then, if the amplitude of the positive RPE generated in the reduced SR system is so large, resulting DA release from such DA neurons might not be able to be fully canceled out by a decrease or pause of DA release from surrounding DA neurons given the asymmetry of the positive and negative phasic responses of DA neurons (Bayer & Glimcher, 2005).

Other than the possible effects of the spiraling RPE information, positive and negative RPEs themselves could cause subjective positive and negative feelings, respectively, given the suggestion that subjective momentary happiness of humans could be explained by reward expectations and RPEs (Rutledge et al., 2014).

### 4.2 Strengths of the present work/model

A strength of our model is that it does not assume drug‐induced direct modulations of the DA system but still considers a key role of DA, and so our model can apply to any kinds of substance or nonsubstance reward and potentially explain the suggested similar involvements of the DA system in addictions to substance and nonsubstance rewards. Habitual, or even addicted, reward taking can arise not only for “DA‐hijacking” substance but also for natural substance, such as food, or nonsubstance, such as gambling, gaming, smartphone use, or relation with other persons. Moreover, it has been suggested that the DA system is also involved in behavioral addiction to nonsubstance reward (Grant et al., 2010). Specifically, there have been suggestions of possible relations of medicines of Parkinson disease to pathological gambling (Dodd et al., 2005; Voon et al., 2006) and of similar changes in the DA system in addiction to substance and nonsubstance such as game (Thalemann et al., 2007) or internet (Hou et al., 2012). In our model, resistance to temptation causes a large positive DA/RPE signal at the rewarded goal in the rigid reduced SR system. Crucially, different from the conventional DA/RPE response to reward, which disappears once the reward becomes predictable, the DA/RPE signal in the rigid reduced SR system continues to be generated. It has thus a similarity to the drug‐induced DA release, providing a potential mechanism for the suggested similar involvements of the DA system in substance and nonsubstance addictions. Previous studies proposed mechanisms for, or applicable to, nonsubstance addiction related to state representation (Redish et al., 2007), high DA release in the nucleus accumbens (Piray et al., 2010), and the complexity of after‐effects (Ognibene et al., 2019). Our proposed mechanism is distinct from, and potentially complementary to, them.

Another, more general strength of the present work lies in its message that inaccurate value estimation due to rigid (inflexible) low‐dimensional state representation, and resulting sustained RPEs that could transmit from one system to another, can potentially lead to behavioral problems and even psychiatric disorders. The SR is a neurally implementable way of partially model‐based RL, but one of its critical drawbacks is policy‐dependence (Momennejad et al., 2017; Piray & Daw, 2019; Russek et al., 2017). Dimension reduction in state representation in the brain is generally suggested (Gershman & Niv, 2010; Niv, 2019), but it is inevitably accompanied by the risk of inaccuracy. The hierarchical cortico‐basal ganglia structure has been suggested to have functional significances (Baladron & Hamker, 2020; Botvinick et al., 2009; Collins & Frank, 2013; Frank & Badre, 2012; Haruno & Kawato, 2006), but it could relate to drug addiction (Keramati & Gutkin, 2013). The present work proposes that a combination of these negative sides can be related to behavioral problems in general, and to addiction in particular.

### 4.3 Drawbacks/limitations of the present work/model

The present model explains why cessation of behavior leading to certain rewards, for which rigid reduced SR has been established, is particularly difficult, but does not explain why rigid reduced SR is formed for some rewards but not others in the first place. We consider that it can depend on the property of reward, duration and frequency of nonresistant reward‐obtaining, and individuals, but exact mechanisms for the formation of rigid reduced SR remains to be addressed. Also, although our model generally points to the empirically suggested similar involvements of the DA system in both substance and nonsubstance addiction, the results of our simulations do not specifically link to known behavioral or physiological results reported for addiction. For this, we will discuss possible neuroimaging experiments in the next section.

Next, our model critically depends on the assumption that the goal‐based reduced SR can be formed in humans and implemented in the brain, but we could not find any direct evidence for them. As for behavioral evidence, we will discuss possible experimental validation in the next section. Regarding neural implementation, we found potentially supporting findings in the literature. Specifically, a finding that the BOLD signal in the ventromedial prefrontal cortex and hippocampus was negatively correlated with the distance to the goal in a navigation task (Balaguer et al., 2016) appears to be in line with such a goal‐based reduced SR; if those regions engaged predominantly in the genuine SR in that task, their overall activity may not show a monotonic increase towards the goal. It is conceivable that the genuine SR can be encoded in the hippocampus (Stachenfeld et al., 2017), but the goal‐based reduced SR can become dominant through intensive training on a particular task or through long‐standing habitual behavior towards a particular goal. Another study (Howard et al., 2014) has shown that the BOLD signal in the posterior hippocampus was positively correlated with the path distance to the goal (increased as the path became farther) during travel periods whereas it was negatively correlated with an interaction between the distance and direction to the goal (increased as the path became closer and more direct) at decision points (and prior studies potentially in line with either of these results are cited therein Morgan et al., 2011; Sherrill et al., 2013; Spiers & Maguire, 2007; Viard et al., 2011)). The goal‐based reduced SR that we assumed can potentially be in line with the activity at decision points, rather than during travel periods, in that study.

Yet another important limitation of the present work is that we modeled the person's resistance to temptation by directly setting the probability of “No‐Go” choice rather than describing the mechanism of action selection (decision making) of the person who has an intention to quit the habitual reward‐obtaining. In terms of value‐based action selection, the Non‐Resistant policy in our model is just optimal, and the Resistant policy is not, unless large punishment is introduced. For this issue, we consider that in addition to the systems for value learning and value‐based action selection/decision making, there would also exist distinct system(s) for rule learning and rule‐based decision making, presumably including prefrontal (especially anterior prefrontal/fronto‐polar) cortical circuits (Miller & Cohen, 2001; Sakai, 2008; Strange et al., 2001). Rule can be set both externally (e.g., by law, or by other person) or internally (as a self‐control). Rule‐based behavior could theoretically be also regarded as a sort of value‐based behavior, driven by punishments (negative values) given when breaking the rules or ethical values emerged when adhering to the rules but can be more absolute or compulsory, and it seems unclear whether such values can also be integrated with other values into a common currency for decision making. Incorporation of the rule‐based system into the model is an important future direction.

### 4.4 Possible experimental validation and clinical implication

The goal‐based reduced SR, the critical assumption of our model, can be considered to be an example of reduced SR where each state is represented by the discounted future occupancies of not all the states but only the states with immediate rewards or punishments; such states themselves could become specifically represented through salience signals. It would be possible to conduct behavioral experiments to examine whether humans adopt such reduced SR or the genuine SR, somewhat similar to the experiments (Momennejad et al., 2017) that compared the reevaluation of reward, transition, and policy. Specifically, if reduced SR based on the states with immediate rewards/punishments is used, adapting to changes in reward placement (i.e., in what states reward is obtained) should be more difficult than adapting to changes in reward size. At the neural/brain level, our model predicts that distinct patterns of RPEs are generated in the systems with the goal‐based reduced SR (Figure 3) and individual (punctate) state representation (Figure 2), which could be reflected in BOLD signals in the striatum where there exist rich DA projections. This prediction can potentially be tested by fMRI experiments and model‐based analyses (Daw, 2011; O'Doherty et al., 2007).

From clinical perspectives, it is essential to know whether the phenomena described by the present model actually occur in people who have a particular difficulty in cessation of long‐standing behavior to obtain reward, and whether the generated RPEs indeed contribute to the difficulty. A potential way is to conduct brain imaging for those people executing a task that simulates their daily struggles against reward‐obtaining behavior, including failures to resist temptation. If it is then suggested that the large positive RPE upon goal reaching generated in the system with the goal‐based reduced SR is an important cause of the difficulty, a possible intervention is to provide alternative reward (physical, social, or internal) upon “No‐Go” decisions expecting that the state representation will change and approach the one under the Resistant policy.

## CONFLICTS OF INTEREST

A.K. is an employee of CureApp, Inc, Japan.

## AUTHOR CONTRIBUTIONS

K.M., K.S., and A.K. conceptualized the study. K.S. conducted the simulations and prepared the graphs. A.K. and K.M. validated the simulations and the graphs. K.M. supervised the project and prepared the original draft. K.S., A.K., and K.M. revised the draft. K.S. and A.K. contributed equally to this work.

### PEER REVIEW

The peer review history for this article is available at https://publons.com/publon/10.1111/ejn.15227.

## Supporting information

Supplementary Material

## Data Availability

Program codes for generating all the data presented in the figures are available in the GitHub (https://github.com/Kshimod/Reduced_SR_RL).
