# Supplementary material for: Rigid reduced successor representation as a potential mechanism for addiction
Source: Eur J Neurosci. 2021 May 10;53(11):3768–90. doi: 10.1111/ejn.15227 (PMC8252639; doi:10.1111/ejn.15227)
Supplement: Supplementary file 1 — Supplementary Material [file EJN-53-3768-s001.pdf]

Rigid Reduced Successor Representation as a Potential Mechanism for Addiction

Kanji Shimomura[1,2+], Ayaka Kato[3,4,5+], & Kenji Morita[1,6*]

[+] These authors contributed equally to this work.

1 Physical and Health Education, Graduate School of Education, The University of Tokyo

2 Department of Behavioral Medicine, National Institute of Mental Health, National Center of Neurology and Psychiatry

3 Department of Life Sciences, Graduate School of Arts and Sciences, The University of Tokyo

4 Laboratory for Circuit Mechanisms of Sensory Perception, RIKEN Center for Brain Science

5 Research Fellowship for Young Scientists, Japan Society for the Promotion of Science

6 International Research Center for Neurointelligence (WPI-IRCN), The University of Tokyo

*Correspondence

Physical and Health Education, Graduate School of Education, The University of Tokyo

7-3-1 Hongo, Bunkyo-ku, Tokyo 113-0033, Japan

E-mail: morita@p.u-tokyo.ac.jp

Supporting Information

For both of the simple RL model with individual (punctate) state representation and the model with rigid goal-based reduced SR of states, we examined the RPEs generated under the Resistant policy in the cases with different parameters. Specifically, we varied the probability of "No-Go" choice ($P_{\text{No-Go}}$) over 0.5, 0.75 (assumed in Figure 2 and 3), and 0.9, and the time discount factor (γ) over 0.95, 0.97 (assumed in Figure 2 and 3), and 0.99. Figure S1, S2, and S3 show the results for RPEs in the 25th episode, over-episode changes of RPEs upon initiation of behavior, and over-episode changes of RPEs at the start state and the goal, respectively, for the simple RL model (panels A) and the model with rigid reduced SR (panels B) (the center panels of Figure S1-3A and S1-3B show the same data as shown in Figure 2C,Da,b and Figure 3E,Ea,b, respectively). As shown in these figures, basic features mentioned in the Results are largely preserved over these parameter ranges.

In order to further examine whether the pattern of RPEs in the model with rigid reduced SR, in particular, the large positive RPE at the goal changes if the learning rate (α_{RSR}), which was set to 0.5 in the simulations shown in the Results, is smaller, we examined the case with α_{RSR} = 0.1. Figure S4 shows the results. As shown in the figure, it took more episodes for the coefficient w and RPEs to become nearly stationary, as expected, but thereafter the generated RPEs look nearly comparable to the case with the original larger learning rate (Figure 3D). This is considered to be because the RPEs primarily come from the sustained mismatch between the true state value function and the estimated value function (i.e., linear function of the features), and the estimated value function on average should not so much depend on the learning rate once learning (probabilistically) converges although the amplitude of within-episode changes of w depends on the learning rate.

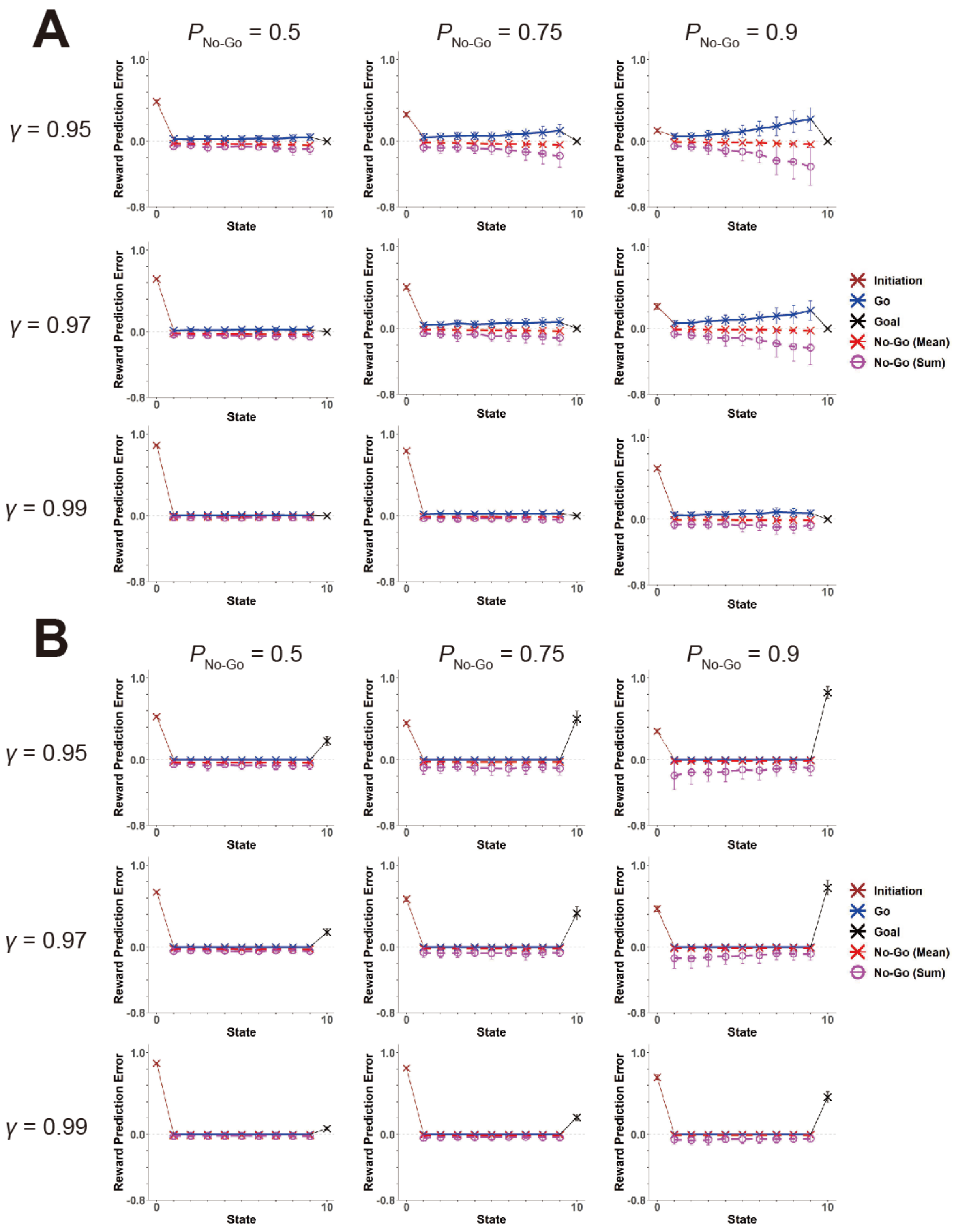

Figure S1

Mean RPEs generated in the 25th episode under the Resistant policy in the simple RL model with individual (punctate) state representation (A) or the model with rigid goal-based reduced SR (B) in the cases with different parameters. Blue: "Go", red cross: "No-Go" mean, magenta circle: "No-Go" sum, brown: initiation of behavior, black: goal. The center panels of (A) and (B) show the same data as shown in Figure 2C and Figure 3D, respectively.

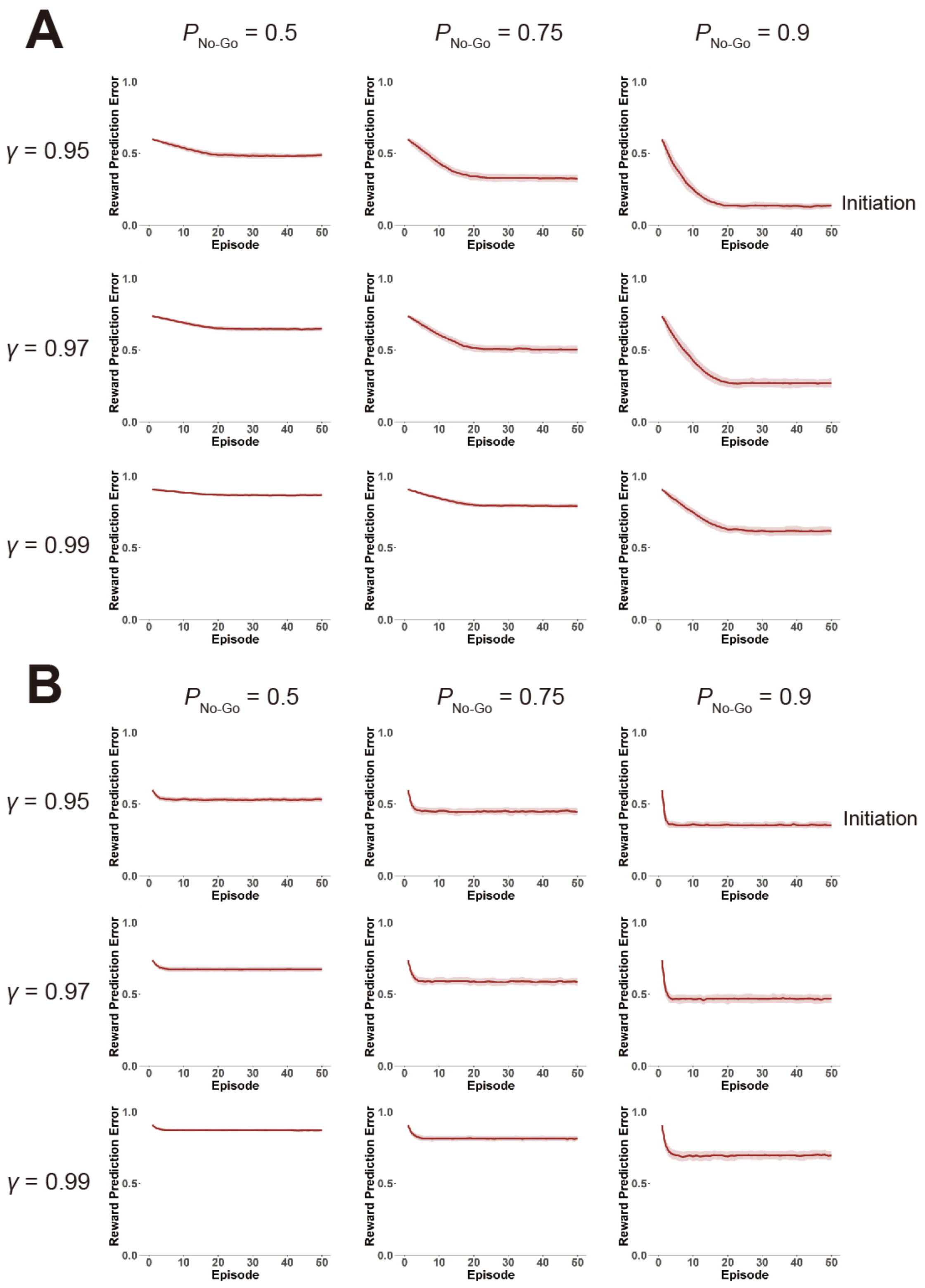

Figure S2

The changes of RPEs upon initiation of behavior over episodes under the Resistant policy in the simple RL model with individual (punctate) state representation (A) or the model with rigid goal-based reduced SR (B) in the cases with different parameters. The center panels of (A) and (B) show the same data as shown in Figure 2Da and Figure 3Ea, respectively.

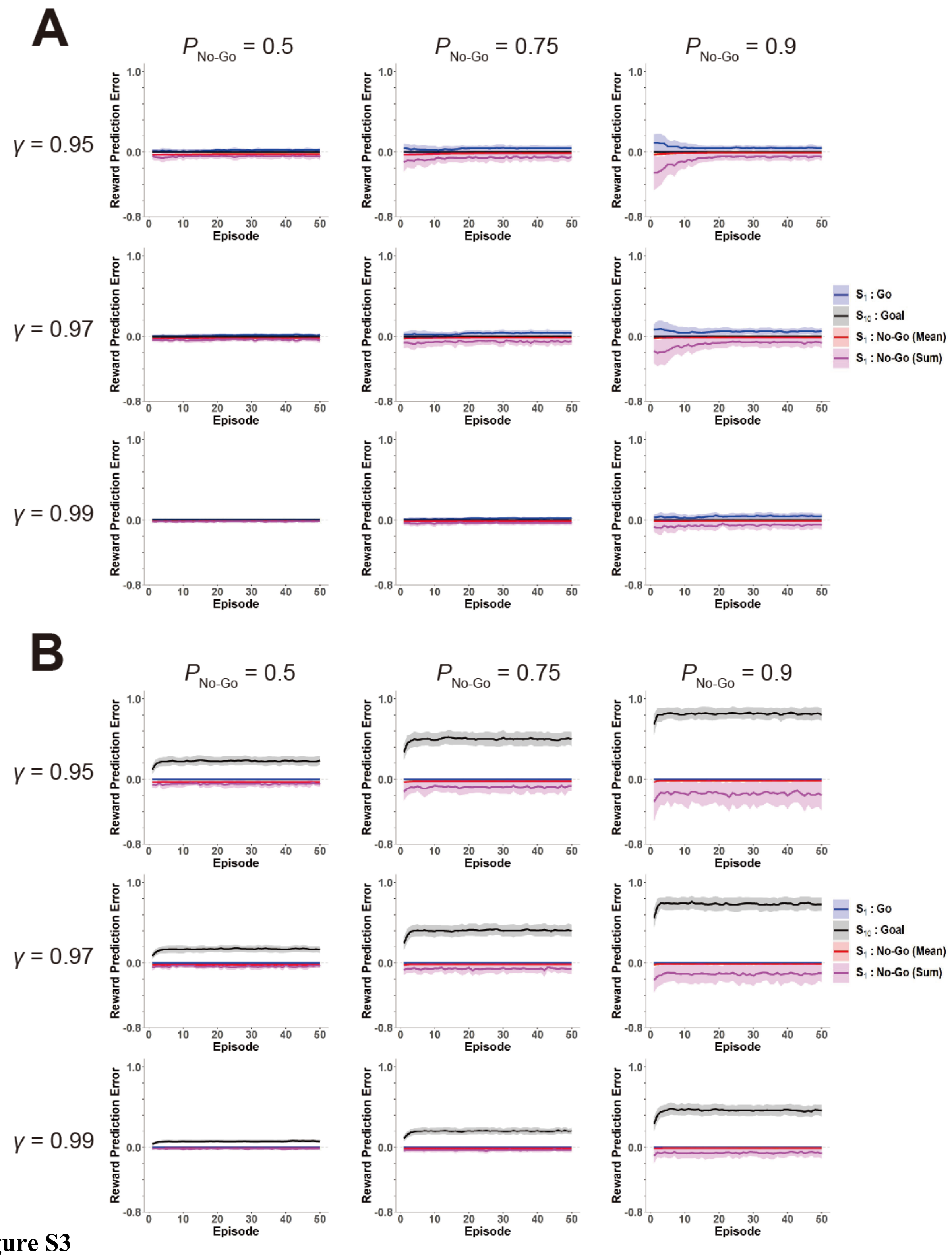

Figure S3

The changes of RPEs at the start state and the goal state over episodes under the Resistant policy in the simple RL model with individual (punctate) state representation (A) or the model with rigid goal-based reduced SR (B) in the cases with different parameters. Blue: "Go" at the start, red: "No-Go" mean at the start, magenta: "No-Go" sum at the start, black: at the goal. The center panels of (A) and (B) show the same data as shown in Figure 2Db and Figure 3Eb, respectively.

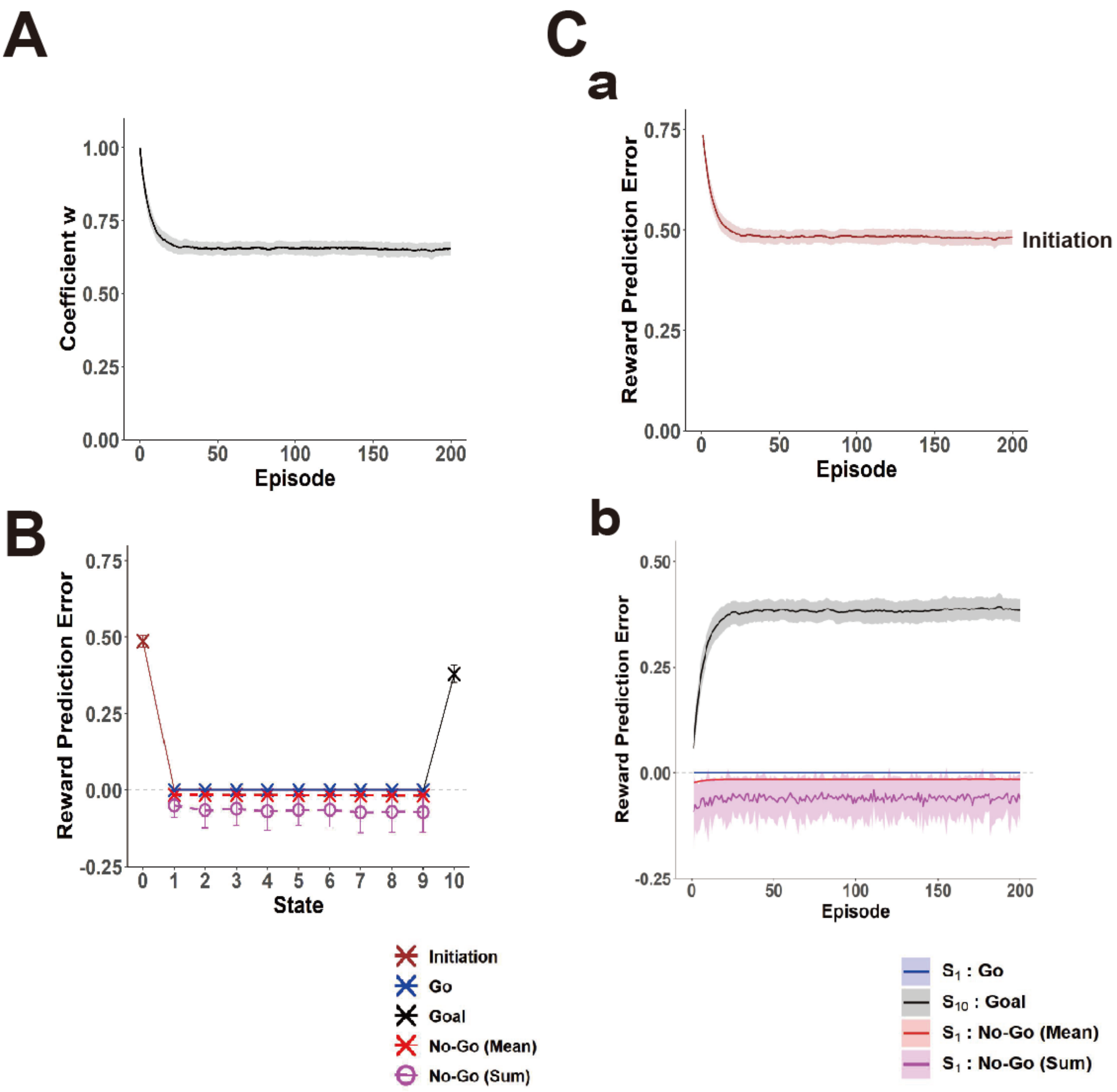

Figure S4

Results of simulations of the model with rigid goal-based reduced SR in the case where the learning rate was smaller ($\alpha_{RSR} = 0.1$) than the original cases ($\alpha_{RSR} = 0.5$). The probability of "No-Go" choice ($P_{No\text{-}Go}$) and the time discount factor (γ) were set to 0.75 and 0.97, respectively (the values assumed in Figure 3). **(A)** Over-episode change of the coefficient w of the approximate value function at the end of each episode under the Resistant policy; the assumed initial value ($w = 1$) is also plotted at episode = 0 with SD = 0. **(B)** Mean RPEs generated in the 25th episode under the Resistant policy. Blue: "Go", red cross: "No-Go" mean, magenta circle: "No-Go" sum, brown: initiation of behavior, black: goal. **(C)** The changes of RPEs over episodes under the Resistant policy. **(a)** RPEs generated upon initiation of behavior. **(b)** RPEs generated at the start state and the goal state.
